# Mutation in *HvCBP20* (*Cap Binding Protein 20*) Adapts Barley to Drought Stress at Phenotypic and Transcriptomic Levels

**DOI:** 10.3389/fpls.2017.00942

**Published:** 2017-06-02

**Authors:** Agata Daszkowska-Golec, Anna Skubacz, Marek Marzec, Michal Slota, Marzena Kurowska, Monika Gajecka, Patrycja Gajewska, Tomasz Płociniczak, Krzysztof Sitko, Andrzej Pacak, Zofia Szweykowska-Kulinska, Iwona Szarejko

**Affiliations:** ^1^Department of Genetics, Faculty of Biology and Environmental Protection, University of Silesia in KatowiceKatowice, Poland; ^2^Department of Microbiology, Faculty of Biology and Environmental Protection, University of Silesia in KatowiceKatowice, Poland; ^3^Department of Plant Physiology, Faculty of Biology and Environmental Protection, University of Silesia in KatowiceKatowice, Poland; ^4^Department of Gene Expression, Faculty of Biology, Adam Mickiewicz University in PoznanPoznań, Poland

**Keywords:** *Hordeum vulgare*, drought, *CBP20*, transcriptome, epidermal pattern, abscisic acid, photosynthesis

## Abstract

*CBP20* (*Cap-Binding Protein 20*) encodes a small subunit of the cap-binding complex (CBC), which is involved in the conserved cell processes related to RNA metabolism in plants and, simultaneously, engaged in the signaling network of drought response, which is dependent on ABA. Here, we report the enhanced tolerance to drought stress of barley mutant in the *HvCBP20* gene manifested at the morphological, physiological, and transcriptomic levels. Physiological analyses revealed differences between the *hvcbp20.ab* mutant and its WT in response to a water deficiency. The mutant exhibited a higher relative water content (RWC), a lower stomatal conductance and changed epidermal pattern compared to the WT after drought stress. Transcriptome analysis using the Agilent Barley Microarray integrated with observed phenotypic traits allowed to conclude that the *hvcbp20.ab* mutant exhibited better fitness to stress conditions by its much more efficient and earlier activation of stress-preventing mechanisms. The network hubs involved in the adjustment of *hvcbp20.ab* mutant to the drought conditions were proposed. These results enabled to make a significant progress in understanding the role of CBP20 in the drought stress response.

## Introduction

ABA signaling under drought stress is extremely complicated and multi-layered. Under drought stress ABA elicits two distinct responses: rapid and gradual. The earliest and most rapid reaction, regulated mainly by ABA, is stomatal closure which minimizes the water loss through limited transpiration. Exposure to ABA triggers guard cells to decrease their volume and close across the airway pore. This is achieved via changes in ion fluxes within the guard cell (Daszkowska-Golec and Szarejko, 2013; Kollist et al., 2014; Munemasa et al., 2015). ABA gradually increases hydraulic conductivity and promotes cell elongation in the root, enabling the plant to recover after water-deficit stress. ABA induces accumulation of osmotically active compounds, which protects cells from damage (Finkelstein, 2013). In 2009, ABA receptors were identified by several teams using different approaches (Ma et al., 2009; Melcher et al., 2009; Miyazono et al., 2009; Nishimura et al., 2009, 2010; Park et al., 2009; Santiago et al., 2009; Yin et al., 2009). Taking advantage from these discoveries the core ABA signaling pathway was established (Hubbard et al., 2010). Since 2009, homologs of Arabidopsis ABA receptors were identified in other species and their role in ABA-mediated drought response was under study (González-Guzmán et al., 2014; Fan et al., 2016).

The recent progress in plant functional genomics has significantly accelerated the process of decoding the ABA-dependent drought stress response in different species (Baek et al., 2010; Park et al., 2015; Sprenger et al., 2016; Zhao et al., 2016). However, further research is still needed to achieve a full understanding of the ABA-dependent drought-response mechanisms and to implement them for the production of crops with an improved drought tolerance.

The negative regulators of ABA signaling serve as promising candidates for such studies since mutations in their genes have often caused a drought-tolerant phenotype in the model species *Arabidopsis thaliana*. Among these genes is *CBP20* (*Cap-Binding Protein 20*), which encodes a small subunit of the cap-binding complex (CBC). CBC is a heterodimer that is formed by two subunits—a small one that is encoded by *CBP20* and a large subunit that is encoded by *CBP80* (*Cap-Binding Protein 80*). Both the nucleotide and amino acid sequences of CBP20 are highly conserved across species from *Saccharomyces* to *Homo sapiens*. CBP20 is involved in very conserved cell processes that are related to RNA metabolism such as polyadenylation and splicing, miRNA biogenesis, and according to the most recent reports, to histone methylation (Kmieciak et al., 2002; Gregory et al., 2008; Kim et al., 2008; Kuhn et al., 2008; Laubinger et al., 2008; Li et al., 2016). Most striking, however, is its simultaneous engagement in ABA signaling during seed germination and drought response (Papp et al., 2004; Jäger et al., 2011). It was shown that an Arabidopsis *cbp20* mutant exhibited a hypersensitivity to ABA during seed germination and a better performance under water deficit conditions than the WT (Papp et al., 2004; Jäger et al., 2011). Interestingly, the Arabidopsis knockout mutant in *CBP80* (*Cap-Binding Protein 80; ABA hypersensitive 1*) exhibited a similar phenotype when exposed to ABA or drought stress (Hugouvieux et al., 2001, 2002; Daszkowska-Golec et al., 2013). In *Solanum tuberosum*, an *amiR80.2-14* mutant (*CBP80* silenced using artificial microRNAs) was also reported to be drought tolerant (Pieczynski et al., 2013).

*H. vulgare* is considered to be one of the leading cereal crops worldwide, and is ranked fourth in terms of harvested acreage and production following maize, rice and wheat (according to the FAO, 2016). The very recent assembling of the 5.1 Gb sequence of the full barley genome (International Barley Genome Sequencing Consortium et al., 2012, 2017) has enhanced the role of barley as a model species for monocotyledonous plants. It should be stressed that *H. vulgare* is the most widely adopted cereal crop that is cultivated under different climatic conditions. Taking into account the negative effects of environmental changes, among them drought stress, on crop productivity and therefore on food availability, it is important to ensure a sufficient quantity and quality of barley yield under adverse environmental conditions (Zhang and Cai, 2011). This can be achieved by implementing effective breeding programs whose aim is to obtain novel cultivars that are characterized by an increased tolerance to stress factors, including drought. However, before implementing any breeding program, basic studies should be performed. A very useful strategy involves the so-called translational genomics approach, which takes advantage of the translation of the data that is obtained in model plants (e.g., *A. thaliana*) on studies of crop species (e.g., *H. vulgare*).

In order to discern the mechanism of *HvCBP20* action under drought stress in barley, we used a unique plant material—a TILLING mutant *hvcbp20.ab* that was developed in our laboratory after chemical mutagenesis. We performed a wide spectrum of analyses that comprised physiological, morphological and transcriptome studies of *hvcbp20.ab* under drought stress. Based on the obtained results, we show new evidence that *HvCBP20* has a pleiotropic effect on the plant traits that results in a better performance under drought stress. The improved response of *hvcbp20.ab* to drought, compared to its wild-type parent “Sebastian,” was manifested by morphological and physiological changes. Analysis of the mutant and WT transcriptomes under control and drought conditions allowed us to gain insight into the molecular regulation of the *hvcbp20.ab* response. These results together with its physiological and morphological traits enabled us to draw a conclusion about the possible mode of action of HvCBP20 under water deficit stress in barley.

## Materials and methods

### Plant material

An *hvcbp20.ab* mutant was isolated using the TILLING (Targeted Induced Local Lesions In Genomes) strategy (McCallum et al., 2000). TILLING was performed in the M_2_ generation of the *Hor*TILLUS population that was developed via the chemical mutagenesis of barley cultivar “Sebastian” in the Department of Genetics, the University of Silesia (Szarejko et al., 2017). The mutated population of ca. 9,600 M_2_ plants were derived from a double treatment of seeds with two mutagens—sodium azide (NaN_3_, 1.5 mM/3 h) and N-methyl-N-nitrosourea (MNU, 0.7 mM/3 h) with a 6-h inter-incubation germination period (iig) between the treatments.

### Mutational analysis of *HvCBP20* using TILLING

**Figure 1:**
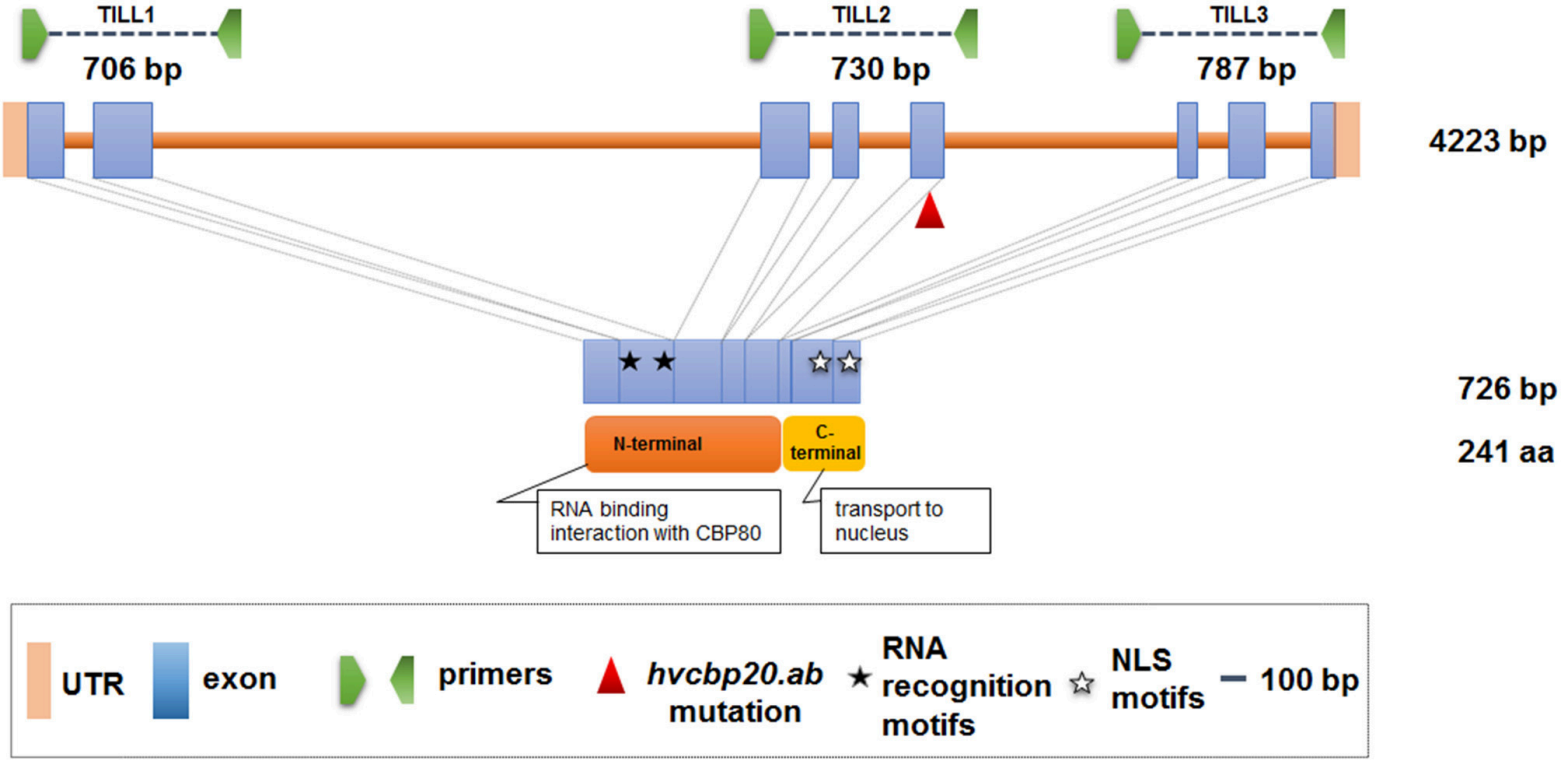
The *HvCBP20* gene structure with the positions of the primers used for the TILLING analysis and *hvcbp20.ab* mutation indicated. Blue box, exon; orange line, intron; pink box, UTR sequences (Untranslated Region); red triangle, *hvcbp20.ab* mutation. The functional domains of the protein: N-terminal responsible for RNA-binding and interaction with CBP80 (Cap-Binding Protein 80); C-terminal—responsible for CBC transport to the nucleus.

Conserved regions of the *HvCBP20* gene were determined using the ClustalOmega (http://www.ebi.ac.uk/Tools/msa/clustalo/) and CODDLE (Codons Optimized to Discover Deleterious Lesions; http://blocks.fhcrc.org/~proweb/input/) tools. Protein functional domains were mapped in a sequence of the *HvCBP20* gene using the Pfam (http://pfam.sanger.ac.uk/) tool. Based on the results obtained, three fragments of the *HvCBP20* gene were selected for TILLING (Figure 1). The first fragment encodes the part of the N-terminal domain that is responsible for RNA-binding and interaction with CBP80 (53–758 bp); the second fragment includes the region that encodes the part of the N-terminal domain that is responsible for the interaction with CBP80 (2,428–3,157 bp) and the third fragment encodes the C-terminal domain that is responsible for the nuclear import of the cap-binding complex (3,435–4,221 bp). The length of three *HvCBP20* gene fragments that were analyzed using TILLING were 706, 730, and 787 bp long, respectively (in total 2,223 bp). DNA pools of eight individual M_2_ plants were used as the templates for DNA amplification. Mutation detection was performed according to the protocol described by Szarejko et al. (2017).

### Computational analysis of the predicted impact of *hvcbp20.ab* mutation on the protein function

The secondary structure was predicted using ENDScript (Robert and Gouet, 2014). The 3D structure of the barley protein (NCBI Acc. No. ACL83596.1) was predicted using LOMETS (http://zhanglab.ccmb.med.umich.edu/LOMETS; Wu and Zhang, 2007) and I-TASSER (http://zhanglab.ccmb.med.umich.edu/I-TASSER; Zhang, 2008; Roy et al., 2010; Yang et al., 2015) servers, which produced similar results. The final 3D structure model was predicted using an LOMETS server based on an *H. sapiens* subunit of CBP20 (1h2nX) as the template. Based on the maximum C-score, the most appropriated predicted structure was chosen for further analysis. The 3D structure was visualized and labeled using UCSF Chimera software (http://www.cgl.ucsf.edu/chimera; Pettersen et al., 2004) running on a Windows system.

In addition, the impact of the substitution that was identified in the *hvcbp20.ab* mutant was analyzed according to the protein affection using the SIFT tool (Kumar et al., 2009). SIFT is a sequence homology-based tool that sorts intolerant amino acid substitutions from tolerant ones. According to the SIFT criteria, the amino acid substitution is predicted to be damaging when the score is ≤ 0.05, and tolerated if the score is >0.05. The median that is used to measure the diversity of the sequences that are used for prediction ranges from 0 to 4.32 (ideally the number is between 2.75 and 3.5).

### Water deficit experiments

#### Water stress treatment

**Figure 2:**
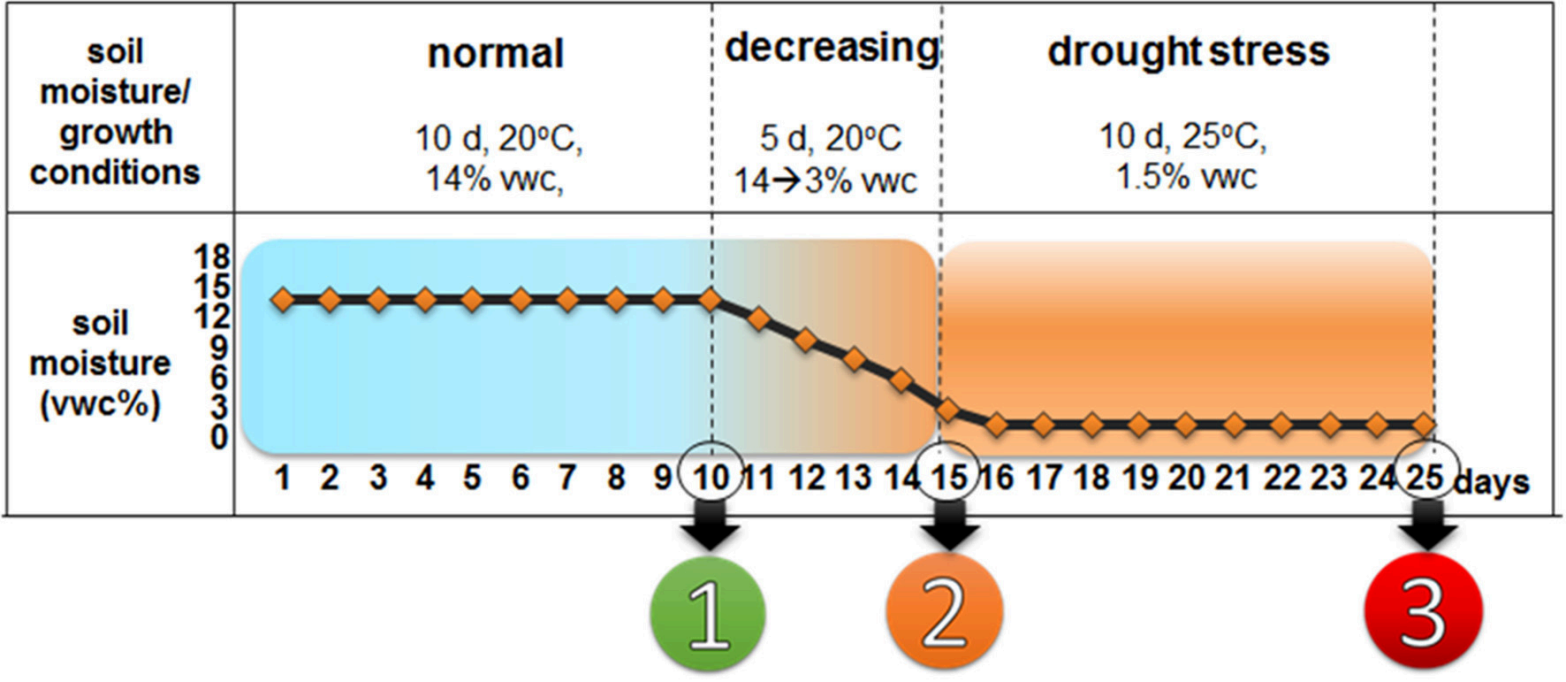
Water-stress experiment performed for the *hvcbp20.ab* mutant and the WT cultivar “Sebastian.” The reference points at which the material was collected and/or analyses were carried out and which are discussed in the text are indicated as: (1) 10 DAS, optimal water conditions, soil moisture of 14%; (2) 15 DAS, soil moisture decreased gradually to 3%, resulting in the onset of drought stress and (3) 25 DAS, soil moisture of 1.5% after 10 d of severe drought.

Water stress was applied in controlled conditions during the seedling stage as described earlier (Kwasniewski et al., 2016; Figure 2). Briefly, boxes (400 × 140 × 175 mm in size) were filled with soil that had known physicochemical properties, which was composed of sandy loam and sand (7:2 ratio) and supplemented with a nutrient medium. In summary, the water-stress experiment included three phases: (i) control growth (14% VWC), which lasted until 10 DAS; (ii) adaptation to the water deficit (3% VWC) which lasted 5 days (11–15 DAS)—also referred to as the onset of drought stress in the text and (iii) water-deficiency stress (1.5% VWC), which lasted 10 days (16–25 DAS)—also referred to as prolonged drought stress in the text (Figure 2). The growth of the control was performed in the presence of a 14% VWC during the entire experiment.

#### RWC analysis

RWC was calculated based on the formula RWC (%) = (F_W_–D_W_)/ (T_W_–D_W_) × 100, where: F_W_ is the fresh weight of the detached second leaf; T_W_ is the turgid weight of the second leaf, which was incubated in distilled water for 24 h in darkness after detachment and D_W_ is the dry weight of the second leaf after it was dried in a dryer at 60°C for 48 h. RWC analysis was performed using three replicates, each consisting of three plants from one box.

#### Chlorophyll *a* fluorescence analysis

Chlorophyll *a* fluorescence was measured using a Plant Efficiency Analyzer (PocketPEA fluorimeter, Hansatech Instruments Ltd., England) as earlier described (Kwasniewski et al., 2016). Measurements of the second leaf of three plants from each of the three boxes were taken as was described above. In the present studies, analysis of chlorophyll *a* fluorescence was based on the JIP-test concept. The OJIP curves were plotted as the relative variable fluorescence, ΔVt, where V_t_ = (F_t_ − F_0_)/(F_m_ − F_0_) according to Strasser et al. (2004) and ΔVt was calculated as the difference between the variable fluorescence values recorded in the control and stressed conditions (Kalaji et al., 2014). Abbreviations of parameters that are used in the text are as follows: F_m_–maximal fluorescence level; F_0_–minimal fluorescence level; F_v_–maximal variable fluorescence; F_t_–fluorescence level at time t; RC/CS–density of RCs (Q_A_–reducing PSII reaction centers); RC–reaction center; CS–cross-section; the OJIP fluorescence induction transient is defined by the names of its intermediate steps: O–fluorescence level at 50 μs; J–fluorescence plateau at 2 ms; I–fluorescence plateau at 30 ms; P–the maximum fluorescence; ABS/RC–absorption flux per active reaction center (RC), DI_0_/RC–dissipation flux per RC, ET_0_/RC–electron-transport flux per RC, PI_ABS_–performance index for the photochemical activity and TR_0_/RC–trapped energy flux per CS (Strasser et al., 2004; Kalaji and Loboda, 2007; Kalaji et al., 2011).

#### Stomatal conductance

The stomatal conductance (mmol m^−2^ s^−1^) of the leaves was determined using an AP4 porometer (Delta-T Devices, Burwell, UK) before midday (referred to the photoperiod in the growth chamber). The measurements were performed in the center of fully expanded second leaves. For each genotype studied, nine leaves (three per replication) were measured on the adaxial side.

#### Electrolyte leakage analysis

The electrolyte leakage was analyzed after 10 days of drought treatment (reference point 3; 25 DAS). The middle part of the leaf (2 cm long) was cut into pieces, washed quickly three times in deionized water, submerged in 20 mL deionized water and kept for 24 h at 10°C. Then, the tube containing the tissue was kept at room temperature in order to adjust the temperature to room temperature and the electrical conductivity was measured using a pH/conductivity meter (CPC-505, Elmetron, Poland) with a glass conductivity cell (EC-60, Elmetron, Poland). Following the conductivity measurement, the tissue was autoclaved for 15 min and cooled to 25°C and the electrical conductivity was measured again. Three replicates (each consisting of the tissues of three plants) were made for the mutant and the WT. The material was treated with a prolonged drought (D) and the control (C). Electrolyte leakage was calculated as the ratios C1/C2 and D1/D2 for the control and drought conditions, respectively, where D1 was the first measurement of drought; D2 was the second measurement of drought; C1 was the first measurement of control and C2 was the second measurement of control.

#### ABA level analysis

The ABA concentration was assayed according to the method described by Nakurte et al. (2012). Chromatographic analysis was performed on a modular HPLC system (Shimadzu, Japan) equipped with a SPD-M20A photodiode array detector and a Kinetex™ C18 (4.6 × 150 mm, 5 μm) column. The injection volume was 20 μl and the analysis was performed in the isocratic mode at a flow rate of 1 mL min^−1^. The results were evaluated using LabSolutions software (Shimadzu, Japan).

#### Epidermal pattern analysis

In order to analyze the density of the stomata and trichome, the SEM technique was used. At reference points no. 1 (10 DAS) and 3 (25 DAS) (Figure 2) during the drought experiment, the material was fixed and prepared as described earlier (Marzec et al., 2013) The microscopic analysis was performed using an SEM (UHR FE-SEM Hitachi SU 8010) at 100x and 1300x magnifications. The measurements were done using ImageJ software (National Institutes of Health; Schneider et al., 2012). The analysis was performed in three biological replicates (three leaves of each of the genotypes studied) with 50 images of 1 mm^2^ of the leaf blade per replication.

#### Monitoring plant growth under different water regimes

Monitoring plant growth during the drought experiment was performed via the non-destructive imaging of the shoot structure as described earlier (Slota et al., 2016). The analysis of the images (JPG or TIFF files) of the plants, which were performed in a photographic chamber, was carried out using ImageJ (National Institutes of Health; Schneider et al., 2012) software that has an implemented macro language to program the operations and to customize the analysis processes. Analysis of plant growth during the water stress experiment was carried out at the reference points throughout the entire experiment: [1]–10 DAS—growth in optimal water conditions; [2]–15 DAS—the onset of drought stress; [3]–22 DAS—after 7 days of drought stress and [4]–25 DAS—10 days of drought stress. A series of photographs of four side views (0°, 90°, 180°, 270°) of the plants in the box were taken. A color reference (green) of known dimension (3 × 3 cm) served as the reference for the calculation of the absolute values of the shoot area. The average values were calculated from each of the side view images (0°, 90°, 180°, 270°). The analyses of the projected shoot area were performed using three biological replicates. One replicate was considered to be one box containing 15 plants per genotype. The height of the plants was measured using three replicates (six plants per replicate).

### Seed germination assay in the presence of ABA

Thirty seeds of the barley cv. “Sebastian” and the *hvcbp20.ab* mutant were planted in a Petri dish plate (Φ = 90 mm) containing four layers of “Whatman” filters that had been soaked with 5 mL of sterile distilled water with or without 100 μM ABA. The plates were chilled at 4°C in the dark for 4 days (stratified) and moved to 22°C with a 16-h-light/8-h-dark cycle. On the second day after stratification (DAS), the Whatman paper filters were replaced with fresh ones. The seeds that were used for these experiments were harvested and stored at the same time. The percentage of seed germination was scored on the 1st, 2nd, 3rd, and 4th DAS. Germination was defined as the visible emergence of the radicle through the seed coat. The analyses were performed using a Stemi 2000-C stereoscopic microscope (Carl Zeiss) with an attached camera (Canon). In order to document the results, AxioVision LE (Carl Zeiss) software was used. The experiment was performed in three biological replicates, with 90 seeds per replicate (each replicate was performed using 3 plates, each containing 30 seeds).

### RNA isolation

The dissected plant tissues were homogenized in a sterile, ice-cold mortar containing 500 μl of an RLT buffer (RNeasy Plant Mini kit; Qiagen, Hilden, Germany). After homogenization, total RNA was extracted using an RNeasy Plant Mini kit according to the manufacturer's instructions. For the microarray analyses, RNA was additionally purified using precipitation in 1 M lithium chloride, and each RNA precipitate was then dissolved in 15 μl of nuclease-free H_2_O. The yield and purity of the RNA was determined using a NanoDrop ND-1000 spectrophotometer (NanoDrop Technologies, Wilmington, USA). The integrity of the RNA was checked using an Agilent 2100 Bioanalyzer equipped with an RNA 6000 Nano chip (Agilent Technologies, Santa Clara, USA).

### Microarray analysis

#### Microarray data analysis

The synthesis, labeling and hybridization of cDNA and cRNA were carried out at the Genomics Core Facility, European Molecular Biology Laboratory (EMBL), Heidelberg, Germany.

The microarray data were analyzed using GeneSpring GX 13.0 software (Agilent Technologies). Hybridization data from all of the biological replicates for each genotype were subjected to per chip normalization using the percentile shift method to the 75th percentile. A baseline transformation was then performed to the median of all of the samples. Statistical testing for differential expression was performed using either the Student *t*-test or a two-way ANOVA followed by the Benjamini-Hochberg false discovery rate (FDR) correction for multiple testing (Benjamini and Hochberg, 1995). A gene was considered to be differentially expressed when the level of its expression between analyzed conditions differed by at least three times ty [fold change (*FC*) ≥ 3; *P* ≤ 0.05 after FDR correction]. The annotation of the Agilent Barley Gene Expression Microarray (Agilent Technologies), which had been done during our previous studies (Kwasniewski et al., 2016), was used for the global analysis of the leaf transcriptomes of the WT cv. “Sebastian” and the *hvcbp20.ab* mutant. The analysis that was performed allowed to use the broad annotations of the high confidence (HC) genes provided by the PLAZA database (http://bioinformatics.psb.ugent.be/plaza/versions/plaza_v3_monocots; Proost et al., 2015), which includes Gene Ontology (GO) annotation, protein domains, homologous gene families and the prediction of orthologous genes. We subsequently used PLAZA for all of the bioinformatics annotations of the barley genes, including an analysis of the barley/Arabidopsis cross-species.

#### GO enrichment analysis

In order to identify the functional ontologies that are associated with the differentially expressed genes (DEGs) and to estimate the enrichment of the functional categories across the treatments, an enrichment analysis based on GO terms was performed using the PLAZA Monocots database version 3.0 (http://bioinformatics.psb.ugent.be/plaza/versions/plaza_v3_monocots; Proost et al., 2015). The GO enrichment tool at PLAZA determines the over-representation of a certain GO term in a gene set compared to the genome-wide background frequency. The significance of over-representation was determined using the hypergeometric distribution followed by the Bonferroni method for multiple testing correction (corrected *P* ≤ 0.01). Additionally, PLAZA was used to identify the corresponding barley orthologs in *A. thaliana* taking into account the extensive annotation features that are available for this species.

#### Quantitative reverse transcription (RT)-qPCR

One microgram of total RNA was used in 20 μl reactions for cDNA synthesis using a Maxima First Strand cDNA Synthesis Kit for RT-qPCR (Thermo Scientific; Waltham, Massachusetts, United States). The cDNA that was obtained was then diluted 1:5 with ddH_2_O and used as the template for the quantitative PCR. All of the primers used in the qPCR were designed using Quant-Prime software (http://quantprime.mpimp-golm.mpg.de/). The 10 μl qPCR reaction mix contained 2 μl of diluted cDNA, 1 μl of the primer pair mixture (5 μM) and 5 μl of 2 × Master Mix (LightCycler 480 SYBR Green I Master; Roche). The following qPCR protocol was used on a LightCycler 480 Real-Time PCR Instrument (Roche) using the SYBR Green I method: initial denaturation for 10 min at 95°C, followed by 45 cycles of 10 s at 95°C, 15 s at 56°C, and 10 s at 72°C, followed by a melting-curve analysis. The reference gene that was used in this study was *EF1* (Elongation factor 1-α; MLOC_3679; Rapacz et al., 2012). Data were analyzed using LinRegPCR (Ramakers et al., 2003) and Excel software (Microsoft). Calculations of the fold change of expression (FC) were done using the formula FC = E^ΔCt^, where E is the mean value of the amplification efficiency of a given gene and ΔCt corresponds to the difference between the mean Ct-values of all of the biological replicates between the two samples that are being compared.

## Results

### Identification of the *hvcbp20.ab* mutation and the prediction of its impact on protein functionality

The functional analysis of *HvCBP20* (Gene Bank Acc. No. FJ548567.1; NCBI) using TILLING was performed on DNA that was extracted from 5,376 plants of the M_2_ *Hor*TILLUS population. In total 11,950,848 bp were scanned and the estimated density of the mutations for *HvCBP20* was 1/373 kb. Three fragments of the *HvCBP20* gene were selected for analysis based on their highest conservation with the orthologous sequences and their engagement in the processes that are crucial for the CBP20 function (Figure 1). A substitution of nucleotide G to A was identified in the *hvcbp20.ab* allele within the gene fragment encoding N-terminal domain responsible for its interaction with the CBP80 subunit. This mutation caused the change of glycine to serine (G141S) in the amino acid sequence.

In order to predict whether an amino acid substitution in a protein affects its functionality, analysis using SIFT (Sort Intolerant from Tolerant; http://sift.jcvi.org/) was performed. According to the analysis, the G141S was predicted to affect the protein function with a score of 0.01. In regards to the SIFT criteria, this substitution is predicted to be damaging. The analysis of the secondary structure of the HvCBP20 that is based on the alignment of orthologs showed a significant difference between barley and human or animal CBP protein and its simultaneous similarity to Arabidopsis and other plants (Supplementary Material S1A). It is worth noting that CBP20 is highly conserved across the compared species when domains responsible for RNA binding-RND (RNA-binding domain) composed of one RNP2 and one RNP1 motif are considered. However, the main difference observed between plant CBP20 and its animal orthologs is the presence of a long C-terminal tail containing nuclear localization signals (Supplementary Material S1A). In Arabidopsis, NLS localized in the C-terminal tail of AtCBP20 is responsible for the import of the whole CBC complex into the nucleus, since the AtCBP80 does not contain NLS (Kierzkowski et al. (2009). Taking into account other highly conserved motifs within the C-terminal tail of plant CBP20 (depicted as green boxes in Supplementary Material S1A), apart from NLSs, it can be hypothesized that the function of this part of CBP20 may be important for activities other than RNA binding and interacting with CBP80 or activities that are specific to plants. The function of these conserved domains is not known yet and needs further investigation.

**Figure 3:**
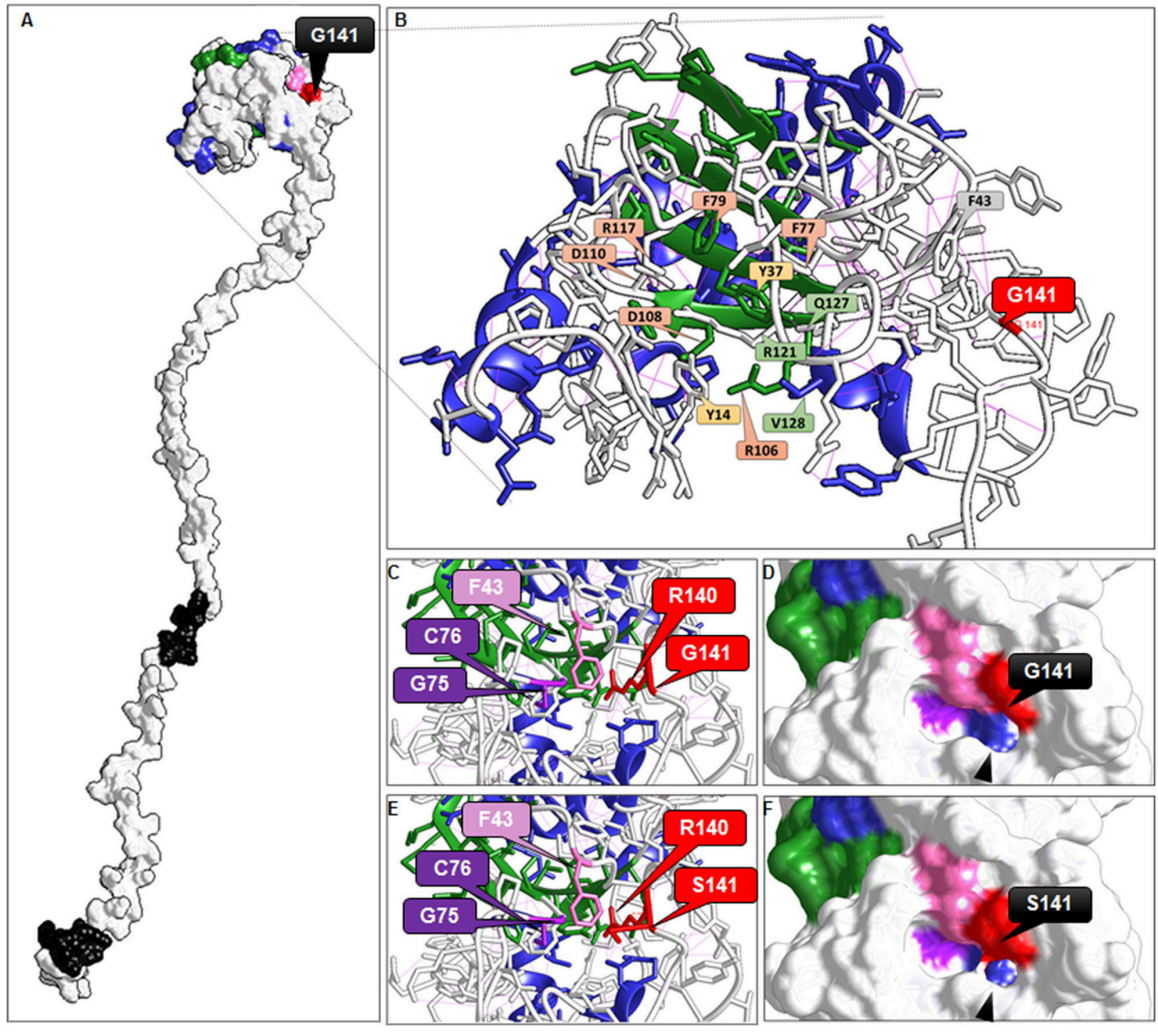
Visualization of the 3D structure of HvCBP20 predicted based on computational modeling using LOMETS and Chimera. **(A)** HvCBP20 protein structure. Black indicates NLS (Nuclear Signal Localization); **(B)** Amino acids responsible for binding the cap and interacting with CBP80 in barley. Based on the analysis of the secondary and tertiary structure of barley CBP20, ortholog alignment and data from Mazza et al. (2001), it was assumed that the cap-binding in barley is performed by Y37, F79, F77, which provide the bottom platform for cap-binding by a salt bridge with R117 and D110. From the top, the Y14 interacts with the ribose on the methylated guanosine of the cap. Two amino acids, D108 and D106, are thought to reinforce these specific interactions. Further, R121 and V128 are responsible for folding the C-terminal domain, whereas Q133 together with R127 initiates the folding of the N-terminal domain through the creation of a salt link with D16; **(C,E)** Comparative modeling of the HvCBP20 region that is crucial for the proper folding of CBP20 **(C)**—the WT while **(E)**—hvcbp20.ab; **(D,F)** WT and hvcbp20.ab differences on the HvCBP20 surface due to G141S mutation.

Then, we attempted to computationally model the *HvCBP20* 3D structure. The 3D modeling confirmed the C-terminal tail and showed that it protrudes from the CBC complex (Figure 3A). Based on the computational analysis, we could visualize the barley CBP20 protein and identify the amino acids that are crucial for RNA binding and interacting with CBP80 (Figure 3). Therefore, we could formulate the assumption that their function might be conserved in barley, Arabidopsis and human homologs. Our comparative analysis allowed us to pinpoint the amino acids that are responsible for binding the cap, interacting with CBP80 and folding the C-terminal domain in barley (Figure 3B). Among these amino acids, which are highly conserved across the species (Supplementary Material S1A), there is F43 (Figures 3C,E), which corresponds to F49 in human CBP20. This amino acid reorients upon cap binding in order to pack between the backbones of the human residues R146-G147 and G81-C82 (Calero et al., 2002; Mazza et al., 2002), which are respectively R140-G141 and G75-C76 in barley CBP20. In the *hvcbp20.ab* mutant, glycine in the 141 position is substituted by serine (G141S). The first observed structure change that was revealed by comparative modeling was the division of the groove that was visible in the wild-type into two parts in the hvcbp20.ab protein (Figures 3D,F). Another change resulting from the G141S mutation was an additional hydrogen bond between the mutated S141 and R140, absent in WT (Supplementary Material S1B). Taking into account the importance of packing the F43 between R140-G141 and G75-C76 for the proper folding of CBP20 protein in humans, it can be presumed that the changes that were observed in the mutated protein may lead to the imperfect folding and thus to its partial dysfunctionality. One can draw a scenario in which the additional hydrogen bond between R140 and S141 interferes with the proper packing of F43 between them and G81-C82, and further affects the proper folding of the CBP20. Taking into account the high conservation of the CBP20 function and the position of the substituted amino acid, the described changes in the hvcbp20.ab structure may be significant for CBP20 functioning. However, these assumptions are based only on computational predictions, and therefore they should be considered to be a hypothesis that needs further verification.

### *hvcbp20.ab* is hypersensitive to ABA during seed germination

**Figure 4:**
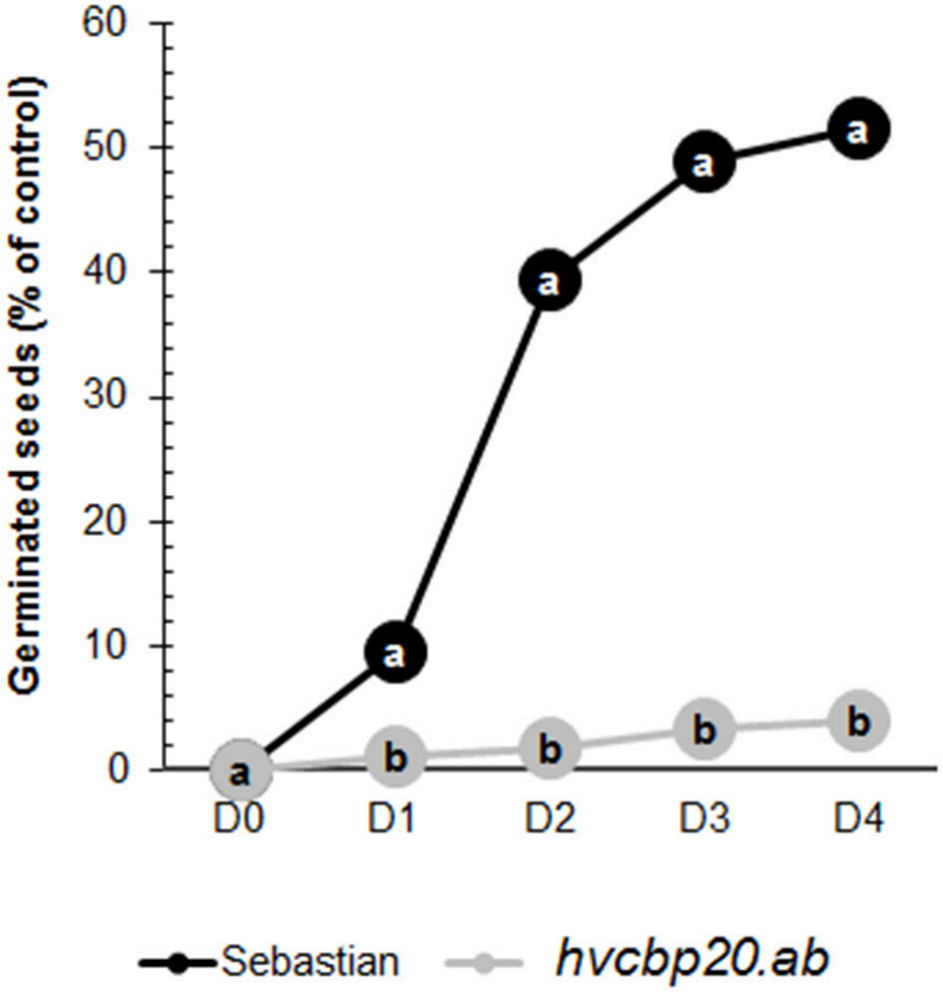
ABA sensitivity assay during seed germination of the *hvcbp20.ab* mutant and WT. WT and *hvcbp20.ab* germination on the medium with 100 μM ABA. Statistical analyses were performed using the *T*-test (*P* < 0.01) to assess the differences between genotypes in the presence of ABA. Statistically significant differences are indicated by different lower case letters. Equivalent means have the same letter.

The *hvcbp20.ab* mutant displayed inhibited seed germination in the presence of 100 μM of ABA. The WT seeds that were with this ABA concentration germinated at a level of 60% of the control (Figure 4). These data support the conservation of the CBP20 function, since the elevated sensitivity to ABA during seed germination of *hvcbp20.ab* is in concordance with studies performed in Arabidopsis *cbp20* and *cbp80* mutants (Hugouvieux et al., 2001, 2002; Daszkowska-Golec et al., 2013). This clearly indicates the engagement of *HvCBP20* in ABA signaling.

### *hvcbp20.ab* shows better performance than its WT parent under drought stress

**Figure 5:**
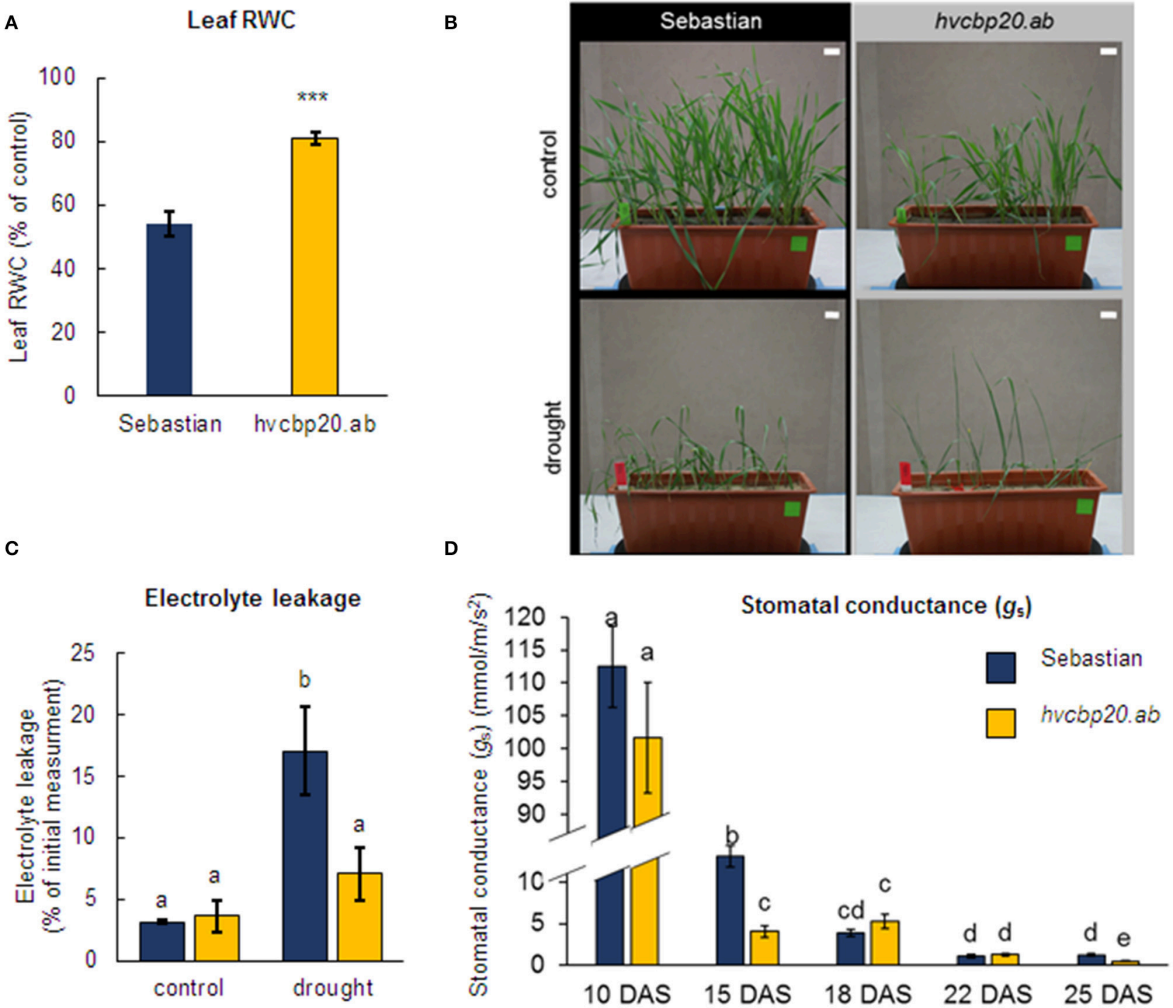
*hvcbp20.ab* response to drought stress. **(A)** Leaf RWC measured in the second leaf of both genotypes studied in control conditions and after 10 days of drought stress treatment. Leaf RWC is presented as % of the control value. Statistical analyses were performed using the *T*-test (^***^P < 0.001) to assess the differences between the genotypes. **(B)** Phenotypes of Sebastian and *hvcbp20.ab* grown in control (top of the panel) and drought (bottom of the panel) conditions. **(C)** Electrolyte leakage measured in the second leaf of both genotypes studied in control conditions and after 10 days of drought stress treatment. Electrolyte leakage values presented on the chart is % of the initial conductivity measurement value. Statistical analyses were performed using two-way ANOVA (*P* < 0.05) followed by Tukey's honestly significant difference test (Tukey HSD-test) (*P* < 0.05) in order to assess the differences between different water regimes and between genotypes. Statistically significant differences (*P* < 0.05) are indicated by different lower case letters. Equivalent means have the same letter. **(D)** Stomatal conductance (gs) measured in the second leaf of both genotypes during the drought stress experiment: 10 DAS—control conditions, 15 DAS—onset of drought stress, 18 DAS and 22 DAS during the drought treatment, 25 DAS—the last day of the 10 days of drought treatment. Each of the values presented are the means ± SE. Statistical analyses were performed using two-way ANOVA (*P* < 0.05) followed by Tukey's honestly significant difference test (Tukey HSD-test) (*P* < 0.05) in order to assess the differences between different water regimes and between genotypes. Statistically significant differences (*P* < 0.05) are indicated by different lower case letters. Equivalent means have the same letter.

The relative water content (RWC) was measured in the second leaf of *hvcbp20.ab* and its WT parent after 10 days of exposure to severe water deficiency stress (25 DAS; Figure 2). There were no significant differences in the RWC between the mutant and the WT under control conditions. After 10 days of drought (25 DAS), the RWC decreased in both of the genotypes studied. However, the mutant was able to store 30% more water than its parent (Figure 5A). The phenotype of drought-stressed plants allowed to distinguish the mutant and the WT genotype. The mutant exhibited leaf rolling and leaves with higher turgid (Figure 5B; Supplementary Material S2A). Another physiological parameter that was highly responsive to drought stress was the electrolyte leakage. After 10 days of drought stress, the WT exhibited a 5.4-fold higher electrolyte leakage than in the control conditions, while the *hvcbp20.ab* did not show any significant differences between the values obtained under a normal water supply and drought (Figure 5C). Taken together, these results indicate an enhanced ability of the *hvcbp20.ab* to store more water and electrolytes within the leaves under water deficiency conditions.

Taking into account that the fastest physiological response of plants exposed to drought stress is stomatal closure (Sirichandra et al., 2009; Tuberosa, 2012), the stomatal conductance (g_s_) during the drought stress was monitored in the *hvcbp20.ab* mutant and its parent cultivar “Sebastian.” Neither genotype differed when stomatal conductance was measured under non-stressed conditions (Figure 5D). At the onset of drought stress (15 DAS), the response of both genotypes was rapid; however, the stomatal closure in the *hvcbp20.ab* was significantly stronger than in the WT. The stomatal conductance in the WT reached the value of the mutant after 3 days of severe drought (18 DAS). No significant changes in *g*_*s*_ between the mutant and “Sebastian” were recorded on the 18 DAS or the 22 DAS. On the last day of drought (25 DAS), both genotypes were characterized by a low stomatal conductance but the mutant exhibited a significantly lower g_s_ than the WT (Figure 5D). It should be noted that the drought tolerance of the *cbp80* and *cbp20* mutants in Arabidopsis was associated with the closure of their stomata (Hugouvieux et al., 2002; Papp et al., 2004). All of these results suggest that the rapid stomatal closure preventing water loss during stress, that was observed in the drought-tolerant CBC mutants, is conserved across species and this universal mechanism is regulated by CBP20/CBP80.

**Figure 6:**
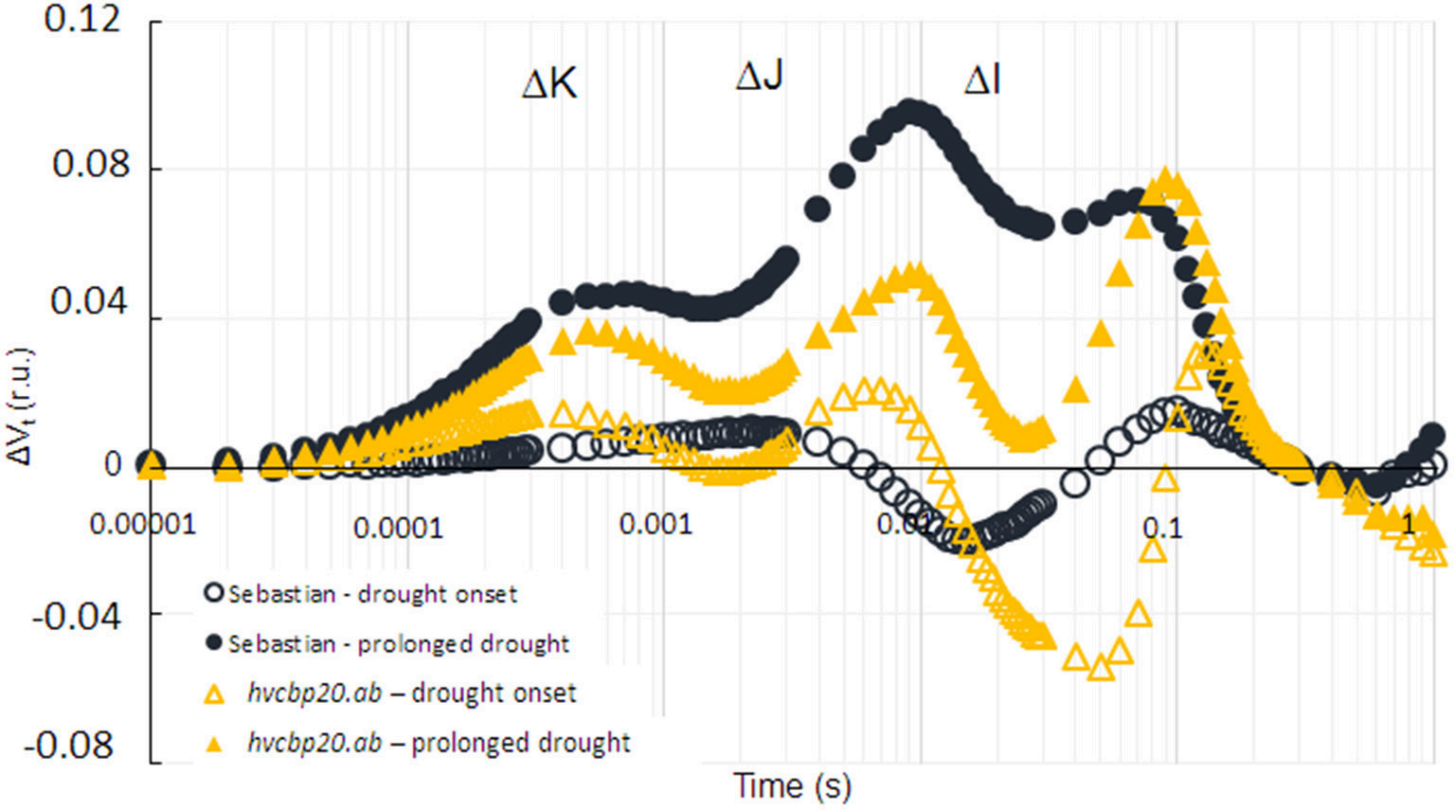
The effect of drought stress on photosynthesis efficiency in *hvcbp20.ab* and cv. “Sebastian.” The effect of drought stress on differential plots of chlorophyll *a* fluorescence (ΔVt) of Sebastian and the mutant. Values are the means. ΔVt curves were constructed by subtracting the normalized fluorescence values (between O and P) recorded in control conditions from those recorded in drought stress.

A decrease in the RWC has been known to induce stomatal closure and thus a parallel decrease in photosynthetic efficiency (Cornic, 2000). To check whether differences between *hvcbp20.ab* and “Sebastian” regarding the state of photosynthetic processes occur under drought stress, the parameters of the JIP-test based on chlorophyll *a* fluorescence were analyzed (Strasser et al., 2004; Zlatev and Yordanov, 2004; Kalaji et al., 2014). The differences between the genotypes studied were revealed during the analysis of the fluorescence transients based on the calculation of the differences in the variable fluorescence curves (ΔV_t_) (Figure 6). In the case of the WT, the curve recorded at the onset of drought stress (15 DAS; depicted as an open blue circles on the Figure 6) did not differ significantly from the control conditions, whereas after the exposure to prolonged drought (25 DAS; depicted as filled blue circles on the Figure 6), it showed a clearly defined Δ*K*-band (300 μs), which was related to the impairment of the oxygen-evolving complex (OEC; Guissé et al., 1995). Moreover, the presence of a high Δ*J*-band (1–2 ms) and Δ*I*-band (10–30 ms) was observed in WT, which were presumed to be associated with an accumulation of ${\text{Q}}_{\text{A}}^{-}$ and the inactivation of ferredoxin-NADP^+^ oxidoreductase (FNR), respectively (Guissé et al., 1995; Schansker et al., 2005). The variable fluorescence curve that was obtained for the *hvcbp20.ab* mutant at the onset of drought (15 DAS; depicted as open yellow triangles on the Figure 6) was characterized by the presence of a low value of each described for WT bands. After 10 days of severe drought stress (25 DAS; depicted as filled yellow triangles on the Figure 6), all of the bands mentioned above were also noted in the mutant, but their values in the mutant were significantly lower for Δ*K* and Δ*J* than the values recorded for the WT under the same conditions.

The OJIP transients were translated into biophysical parameters according to Strasser et al. (2004)—specific activities per reaction center (RC) and performance index (PI_ABS_; Table 1). Changes in parameters of photosynthesis in the mutant were observed already at the onset of drought stress, while WT exhibited changed photosynthetic parameters only after prolonged drought (Table 1). Interestingly, most of the parameters measured in *hvcbp20.ab* mutant at the onset of drought stress were not significantly different from the WT-values recorded under prolonged drought. Moreover, the values of ABS/RC, TR_0_/RC, ET_0_/RC, and DI_0_/RC were not significantly changed in the period between 15 DAS until the 25 DAS in the mutant, except for RC/CS_0_ parameter (Table 1). At the onset of drought (15 DAS), the increased value of absorbed energy (ABS/RC) was observed in *hvcbp20.ab*. This sort of reaction can be related to either (i) the inactivation of a part of the RC pool or (ii) increased size of the antenna in response to stress conditions (Kalaji et al., 2014). The analysis of the number of active RCs per excited cross section (RC/CS_0_) revealed that the cause of changes in ABS/RC in the mutant was a significant reduction of reaction centers at the onset of drought. The increase of trapping energy per active reaction center (TR_0_/RC) according to literature (Kalaji et al., 2014) is associated with changes in ABS/RC and RC/CS. The same relation was observed at the onset of water deficiency in the mutant and after the prolonged drought in the WT. It is worth noting that this parameter (TR_0_/RC) is tightly linked to the appearance of the Δ*K* band, since it is directly related to the impairment of the oxygen-evolving complex (OEC). Taking these results together, the photosynthetic machinery of the mutant adapted to the water deficit faster than the WT.

**Table 1:** Effect of drought stress on the “Sebastian” and *hvcbp20.ab* plants analyzed using the JIP-test.

**Parameter**	**Sebastian**			***hvcbp20.ab***		
	**control**	**drought onset**	**Prolonged drought**	**control**	**drought onset**	**Prolonged drought**
ABS/RC	1.70 ± 0.04^a^	1.70 ± 0.05^a^	2.03 ± 0.09^b^	1.80 ± 0.04^a^	2.10 ± 0.08^b^	2.10 ± 0.06^b^
TR_0_/RC	1.35 ± 0.03^a^	1.36 ± 0.04^a^	1.57 ± 0.02^b^	1.40 ± 0.02^a^	1.54 ± 0.04^b^	1.57 ± 0.03^b^
DI_0_/RC	0.35 ± 0.01^a^	0.36 ± 0.01^a^	0.46 ± 0.02^b^	0.44 ± 0.02^b^	0.53 ± 0.03^c^	0.52 ± 0.03^c^
ET_0_/RC	0.89 ± 0.02^a^	0.89 ± 0.02^a^	0.96 ± 0.03^b^	0.90 ± 0.01^a^	0.99 ± 0.02^b^	0.96 ± 0.02^b^
RC/CS_0_	241.0 ± 4.0^a^	242.0 ± 3.9^a^	206.4 ± 9.6^c^	250.0 ± 2.6^a^	222.3 ± 2.5^d^	183.9 ± 7.2^b^
PI_ABS_	4.25 ± 0.213^c^	4.00 ± 0.162^c^	2.78 ± 0.15^ab^	3.17 ± 0.223^b^	2.69 ± 0.27^ab^	2.38 ± 0.21^a^

*In the table, the means ±SE are presented for each of the parameters that were analyzed. Statistical analyses were performed using two-way ANOVA (P < 0.05) followed by the Tukey honest significant difference test (Tukey HSD-test) (P < 0.05) to assess the differences between the treatments (control, drought onset, and prolonged drought) within a genotype and between genotypes. Statistically significant differences (P < 0.05) are indicated by different lower case letters (a, b, c, d). Equivalent means have the same letter. A decrease (in relation to control) in a parameter is indicated in red and an increase in green. A gradual decrease of a parameter from the onset of drought until the end of the prolonged drought is indicated in orange first and then in red (e.g., RC/CS_0_ in mutant); ABS/RC, absorption flux per active reaction center (RC); DI_0_/RC, dissipation flux per RC; ET_0_/RC, electron-transport flux per R; PI_ABS_, performance index for the photochemical activity; TR_0_/RC, trapped energy flux per CS*.

Our results indicated also that *hvcbp20.ab* was able to shift the excess of light energy into dissipation more effectively and much earlier that its WT parent. At the onset of drought, the *hvcbp20.ab* mutant had already achieved 120% of the control DI_0_/RC-value and interestingly, it still displayed the same dissipated energy value after prolonged water deficiency (Table 1). Conversely, cv. “Sebastian” did not dissipate more energy at the onset of drought compared to the control conditions but after 10 days of drought stress, the DI_0_/RC increased by 30% compared to the control value. These results together indicated a faster adjustment of the action of the photosynthetic apparatus in the *hvcbp20.ab* mutant.

An analysis of growth reduction under drought stress revealed that the mutant did not react as drastically to a water deficiency as its WT (Table 2). A significant reduction of *hvcbp20.ab* growth (93% of the height of the control plants grown under optimal conditions) was observed only on the last day of drought treatment (25 DAS), whereas a reduced growth rate of Sebastian (67% of the growth of the control) was observed already after 5 days of severe drought (22 DAS; Table 2). The prolonged drought stress did not lead to such a rapid reduction in growth rate in *hvcbp20.ab* as it did in WT. This may be related to the strong increase in the ABA level in the mutant at the onset of drought stress (about 100% more than the control conditions), whereas the highest peak of the ABA level in the WT occurred only after prolonged drought treatment (Supplementary Materials S3A,B). These results provide additional evidence of a delayed response to drought stress in the WT. Non-destructive imaging of plants during the drought experiment showed that the mutant produced a significantly larger (20% over the wild-type) projected shoot area than its wild-type at the beginning of drought stress (15 DAS; Supplementary Material S2B). However, in accordance with the expectations, the projected shoot area in both genotypes decreased during the drought treatment, and *hvcbp20.ab* exhibited a significantly larger projected shoot area (7% more than the wild type; Supplementary Materials S2B,C) at the end of the treatment. Taking into account that the traits that were observed in *hvcbp20.ab* such as leaf rolling and thus its later turgid loss were also reported to be associated with a later onset of the senescence process (Neilson et al., 2015), it can be concluded that the larger shoot area may be a common outcome of these events.

**Table 2:** Comparison of growth rate under the control and drought stress treatment of the “Sebastian” and *hvcbp20.ab* plants.

**Day after sowing (DAS)**	**Height of plant (cm)**			
	**Control conditions**		**Drought conditions**	
	**Sebastian**	***hvcbp20.ab***	**Sebastian**	***hvcbp20.b***
10	11.3 ± 0.6^b^	8.1 ± 1.4^a^	-	-
15	23.4 ± 2.0^b^	18.7 ± 2.3^a^	21.8 ± 1.4^b^	18.3 ± 3.7^a^
			93%	97%
22	28.8 ± 3.1^c^	21.8 ± 3.5^ab^	19.4 ± 2.4^a^	22.1 ± 2.7^b^
			67%	101%
25	29.8 ± 4.3^c^	23.0 ± 2.9^a^	22.8 ± 2.5^a^	20.2 ± 3.5^b^
			76%	93%

*Comparison of the height (cm) of the hvcbp20.ab and “Sebastian” plants under the control (optimal water supply) and drought conditions. In the table, the means ±SE are presented. Statistical analyses were performed using two-way ANOVA (P < 0.05) followed by the Tukey honest significant difference test (Tukey HSD-test) (P < 0.05) to assess the differences between the genotypes under control and drought conditions on the indicated day of the experiment. Statistically significant differences (P < 0.05) are indicated by different lower case letters (a, b, c, d). Equivalent means have the same letter. Percentage values are the % of the control growth of each genotype on the indicated day after sowing (DAS). The height of a plant is expressed in cm*.

### The epidermal pattern of *hvcbp20.ab* leaves revealed the specific mechanism of its adaptation to drought

**Figure 7:**
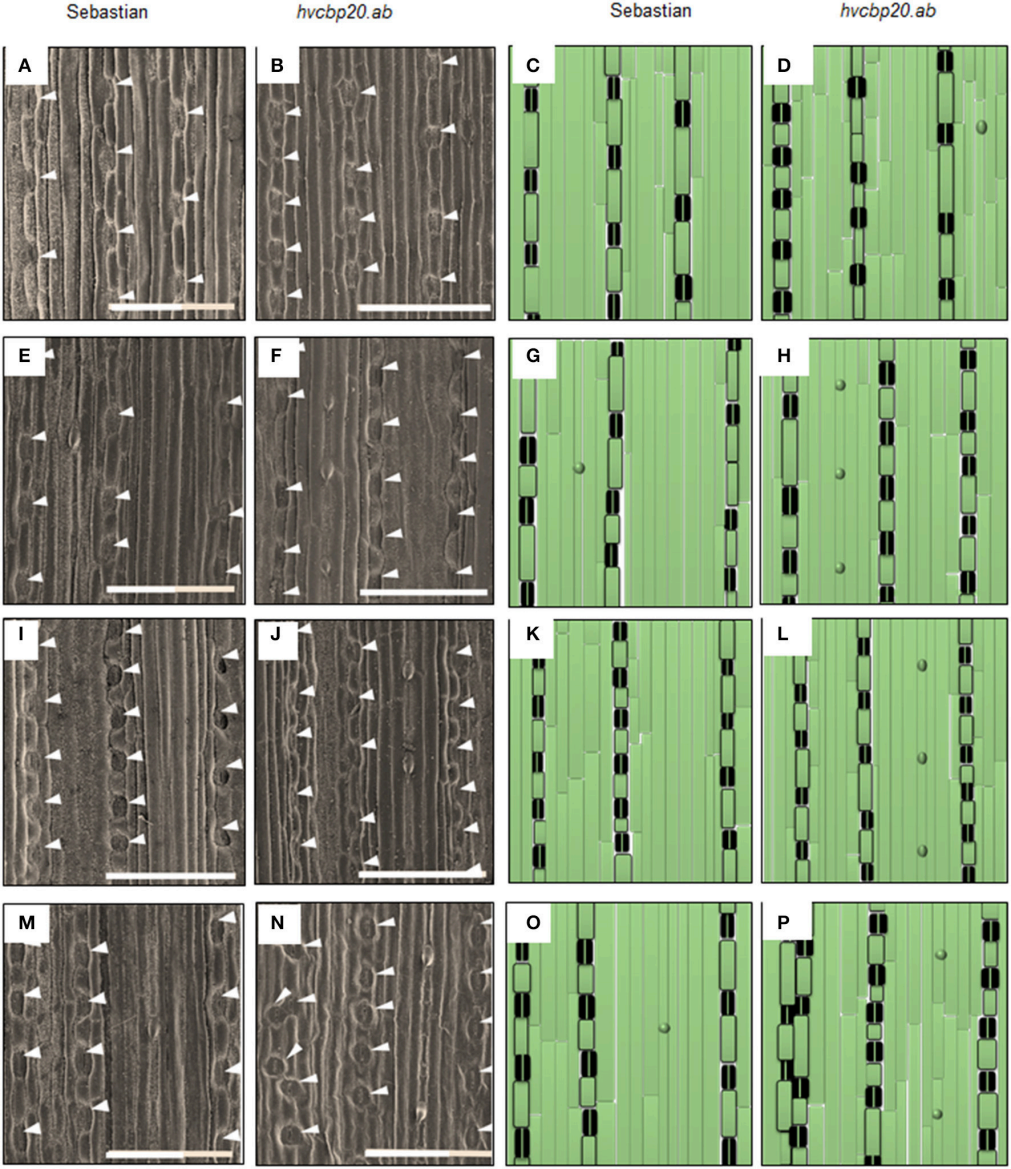
Epidermal pattern in “Sebastian” and *hvcbp20.ab*. **(A–D)** Epidermal pattern on abaxial surface of the leaf in control conditions; **(E–H)** Epidermal pattern on abaxial surface of the leaf after 10 days of drought treatment; **(I–L)** Epidermal pattern on adaxial surface of the leaf in control conditions; **(M–P)** Epidermal pattern on adaxial surface of the leaf after 10 days of drought treatment. The microscopic analysis was performed using SEM (UHR FE-SEM Hitachi SU 8010) under the 100x magnification. Analysis was performed in three biological replicates (three leaves of each genotype studied). The size of analyzed field was 1 mm^2^ and 50 images of the same part of each of the three leaf blades were analyzed per genotype. The bar = 200 μm.

Analysis of the epidermal cell pattern in *hvcbp20.ab* revealed that the mutant differed from the WT parent under control conditions (Table 3; Figure 7). This morphological trait may be one of the phenotypic modifications that ensure the mutant's adaptation to drought. In non-stressed conditions, *hvcbp20.ab* exhibited 9% fewer stomata and 26% more trichome-like structures on the adaxial surface of the epidermis than the WT parent. It should be noted that the guard cells of mutant were smaller, more precisely shorter (80% of the WT) and wider (124% of the WT). These results are in accordance with previously reported *cbp80* mutants of *A. thaliana* and *S. tuberosum*, which exhibited fewer stomata (respectively, 75 and 80% of the WT) and more trichomes (respectively, 140% and 130% of the WT) on the adaxial side of the leaves (Daszkowska-Golec et al., 2013; Pieczynski et al., 2013).

**Table 3:** Differences in the epidermal pattern of “Sebastian” and *hvcbp20.ab*.

**Trait**	**Adaxial surface of the leaf**				**Abaxial surface of the leaf**			
	**Control**		**Drought**		**Control**		**Drought**	
	**Sebastian**	***hvcbp20.ab***	**Sebastian**	***hvcbp20.ab***	**Sebastian**	***hvcbp20.ab***	**Sebastian**	***hvcbp20.ab***
No. of guard cells (GC)	85.6 ± 2.6^c^	76.3 ± 2.7^b^	67.9 ± 2.7^a^	69.0 ± 4.0^a^	73.5 ± 0.9^b^	77.8 ± 1.5^c^	62.2 ± 1.9^a^	82.7 ± 1.8^d^
Av. length of GC (μm)	35.2 ± 0.5^a^	31.1 ± 0.8^b^	39.0 ± 1.1^c^	34.3 ± 0.7^a^	35.5 ± 0.6^a^	32.3 ± 0.9^a^	40.1 ± 0.6^c^	36.8 ± 0.2^b^
Av. width of GC (μm)	16.1 ± 0.4^b^	19.9 ± 1.3^a^	20.4 ± 0.9^a^	23.2 ± 0.8^c^	14.2 ± 0.4^b^	24.0 ± 0.5^c^	18.3 ± 0.4^a^	19.2 ± 0.4^a^
Av. length of cells between GC (μm)	54.8 ± 1.0^a^	59.1 ± 2.1^a^	72.4 ± 1.6^b^	57.2 ± 2.8^a^	68.1 ± 2.4^a^	58.3 ±0.8^b^	94.6 ± 1.2^c^	65.0 ± 0.6^a^
No. of trichomes	15.4 ± 1.5^ab^	19.5 ± 1.6^c^	13.4 ± 1.1^a^	16.4 ± 1.0^bc^	15.8 ± 0.8^b^	18.9 ± 0.6^c^	9.6 ± 1.0^a^	23.3 ± 0.2^d^

*In the table, the means ± SE are presented for each of the traits that were analyzed. Statistical analyses were performed using two-way ANOVA (P < 0.05) followed by Tukey's honest significant difference test (Tukey HSD-test) (P < 0.05) to assess the differences between the genotypes (Sebastian and hvcbp20.ab) and the treatments (control and prolonged drought) in the epidermal pattern on adaxial or abaxial surface of a leaf. Statistically significant differences (P < 0.05) are indicated by different lower case letters (a, b, c, d). Equivalent means have the same letter. A decrease (in relation to control) of a parameter is indicated in red and an increase in green*.

The abaxial surface of the *hvcbp20.ab* leaves had significantly more (10%) guard cells than the WT, and the guard cells were smaller, similar to those that were observed on the adaxial side (Table 3). Additionally, the abaxial surface of the mutant leaf was coated with 20% more trichome-like structures than the WT (Table 3; Figure 7). Our observations were similar to those of a study that was performed in a *cbp20* mutant of *A. thaliana* in which more guard cells that were smaller in size together with more trichomes than in the wild-type were observed (Jäger et al., 2011). Taken together, the epidermal pattern of *hvcbp20.ab* under the control conditions indicates its potential adaptation to stress conditions—the lower number of stomata on the adaxial side, which is more exposed to adverse environmental conditions and the increased number of stomata on the abaxial side of the leaf together with an increased number of trichome-like structures. All of these traits help to balance the transpiration rate.

However, the most striking observation was the plasticity of the epidermis pattern in response to drought conditions in the *hvcbp20.ab* mutant. It has already been shown that changes in the number of guard cells are induced in response to changes in the environmental conditions (Yoo et al., 2010). The most common response is a reduction in the number of guard cells. The total number of stomata was also reduced on the adaxial side of the leaf in both genotypes studied in response to drought (Table 3). It is worth noting that after drought treatment, the length of the cells separating the guard cells was increased by 30% in the WT, whereas no change was observed in *hvcbp20.ab* (Table 3; Figure 7). Interestingly, the abaxial side of the leaf in the mutant after drought exposure was characterized by an increased number of stomata (132% of the WT) that were smaller. In addition, the cells separating the guard cells were shorter than in the wild type under the same conditions (60% of the wild type; Table 3; Figure 7). Additionally, the abaxial side of the epidermis in the mutant was coated with an increased number of trichomes (240% of the wild type; Table 3; Figure 7). Taken together, our observations allowed us to propose the model of the *hvcbp20.ab* leaf that describes changes in epidermal pattern in order to adapt to drought. We speculate that when the mutant leaf was rolled, it formed a humid environment and thus enabled minimal transpiration even under drought stress conditions. On the other hand, the abaxial side of the leaf in the mutant after drought exposure was enriched with increased number of smaller stomata, coated with an increased number of trichomes and also the cells separating the guard cells were shorter than in the wild type under the same conditions (Supplementary Material S4).

### Transcriptomic analysis of the *hvcbp20.ab* mutant and its wild type

With the aim to examine the molecular mechanism of *hvcbp20.ab* response to water deficiency, the genome-wide transcriptome analysis was performed. We have already applied Agilent microarray with success for evaluation of transcriptome changes induced by drought in a root hairless mutant and its parent cultivar (Kwasniewski et al., 2016). With the aim of testing the reliability of the results that were obtained with the microarray, the expression of differentially expressed genes (DEGs) was validated using sensitive RT-qPCR (see Supplementary Material S5).

#### Analysis of *hvcbp20.ab* and “sebastian” transcriptomes in optimal water conditions

**Figure 8:**
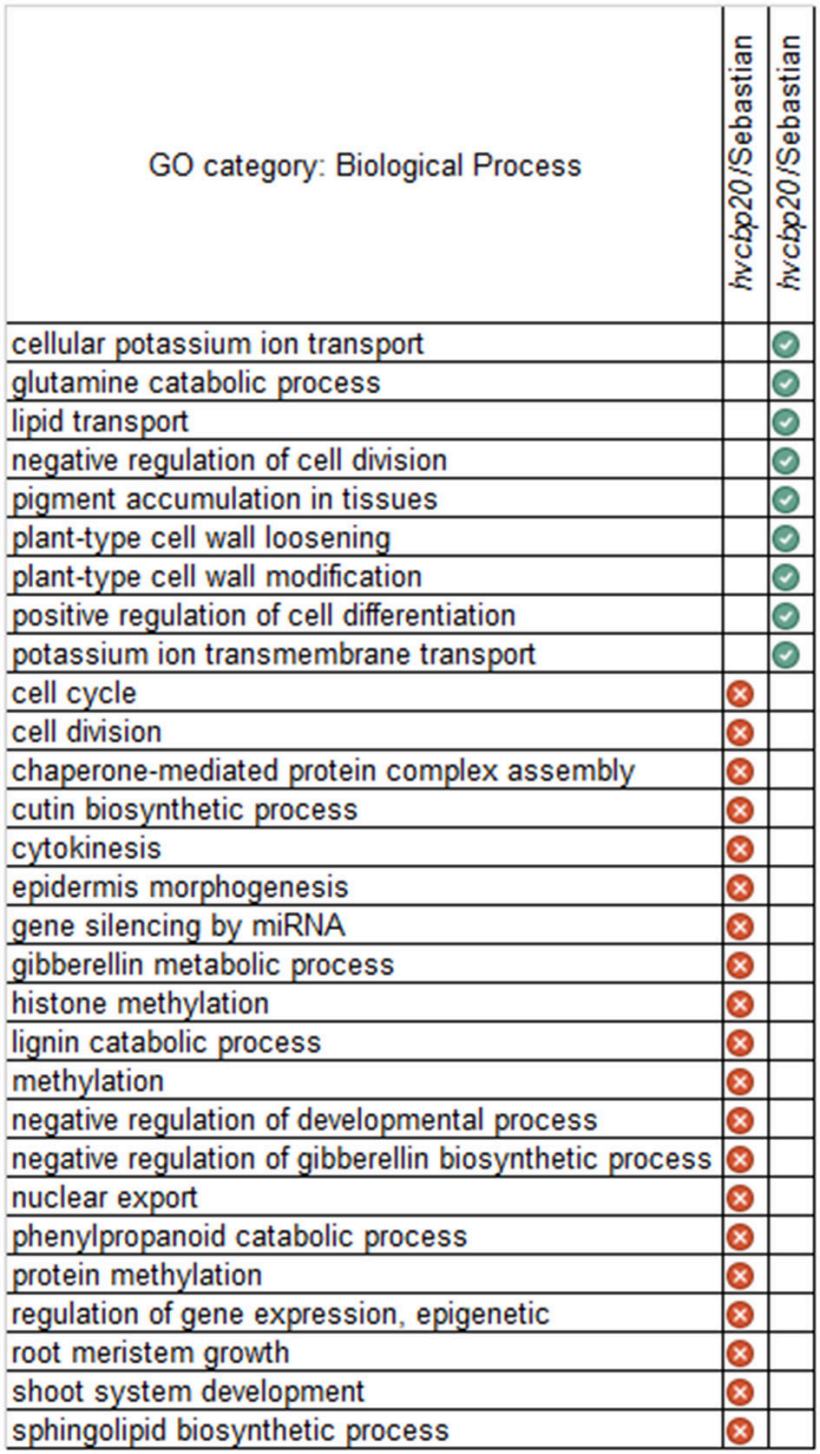
GO categories (Biological Processes) that were over-represented in the *hvcbp20.ab* mutant when compared to the “Sebastian” in control conditions. BPs that were over-represented in the subsets of genes: down-regulated genes are indicated by a white “x” inscribed in a red circle and up-regulated genes are indicated by a white tick inscribed in a green circle (corrected *P* < 0.01).

We have started the transcriptome analysis by comparing transcriptomes of *hvcbp20.ab* and “Sebastian” seedlings that were grown under optimal water supply. The differential analysis of transcriptomes led to the identification of 127 up-regulated and 327 down-regulated HC genes in *hvcbp20.ab* in relation to its wild type (*FC* ≥ 3; *P* ≤ 0.05 after FDR correction, Supplementary Material S6). In order to gain an insight into biological role of identified differentially expressed genes (DEGs), the functional annotation was performed using the PLAZA tools (http://bioinformatics.psb.ugent.be/plaza/). The analysis performed for the set of genes up-regulated in *hvcbp20.ab* in relation to its WT parent showed involvement of *HvCBP20* in processes such as: negative regulation of cell division, positive regulation of cell differentiation, plant-type cell wall loosening, cellular potassium ion transport and lipid transport (Figure 8; Supplementary Material S6). Most of Biological Processes (BP) that represented genes down-regulated in *hvcbp20.ab* pointed to processes related to RNA metabolism and epigenetic modifications (Figure 8; Supplementary Material S6). Taking these results together, our analysis clearly showed that the identified *hvcbp20.ab* mutation led to the disruption of processes linked with CBC action described earlier for humans and Arabidopsis (reviewed in Gonatopoulos-Pournatzis and Cowling, 2014). Interestingly, the engagement of CBP20 in the methylation process, which was indicated by our results, has been recently proven by Li et al. (2016) in Arabidopsis study.

#### Comparative analysis of *hvcbp20.ab* and “sebastian” transcriptomes under drought stress

The comparative leaf transcriptome analysis of “Sebastian” and *hvcbp20.ab* at three reference points including: (1) control growth under optimal water conditions (10 DAS), (2) the onset of drought stress (15 DAS) and (3) prolonged drought stress lasting 10 days (25 DAS) was performed. The microarray data analysis was conducted as a calculation of the differential expression of genes within a genotype between subsequent reference points (2-1 and 3-1).

**Figure 9:**
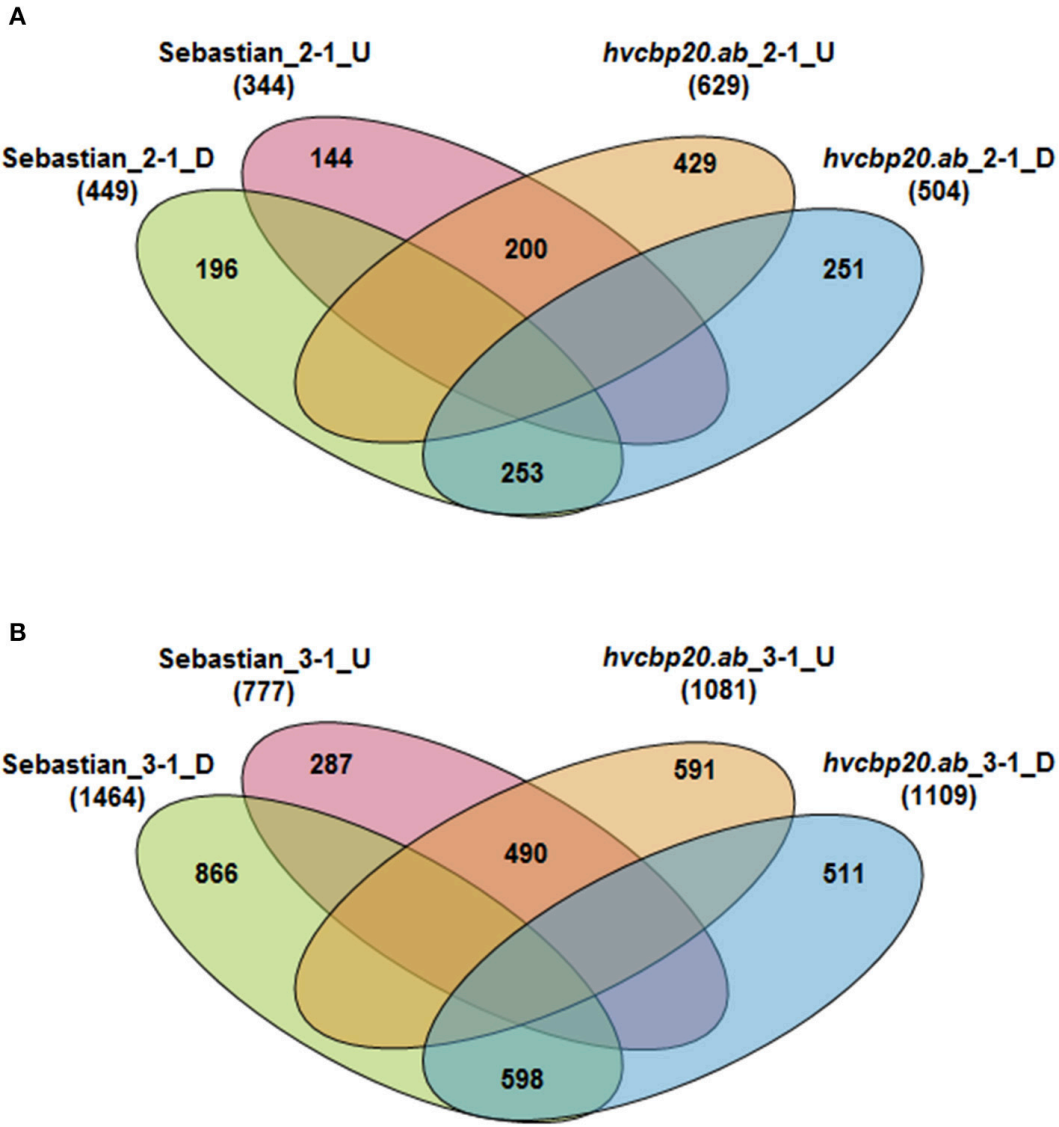
Comparative analysis of the numbers of DEGs during the drought experiment in the WT cv. “Sebastian” and the *hvcbp20.ab* mutant. **(A)** Comparative analysis of the number of DEGs at the onset of drought stress. **(B)** Comparative analysis of the number of DEGs after prolonged drought stress. In the Venn diagrams, the subsets of the genes that were up-regulated or down-regulated specifically in “Sebastian” or the *hvcbp20.ab* mutant during the subsequent stages of the experiment in comparison with the control conditions are indicated (*P* ≤ 0.05 after FDR correction; *FC* ≥ 3).

Detailed analysis of the list of genes that were regulated by water stress revealed differences in response to drought between *hvcbp20.ab* mutant and its wild-type. Overall, 1,473 and 3,343 high confidence (HC) genes were differentially regulated at the drought onset and during the prolonged drought treatment, respectively (*P* ≤ 0.05 after FDR correction; *FC* ≥ 3). Interestingly, 2-fold more genotype-specific genes responded differentially in mutant (680) than in the wild type (340) during the initial phase of the experiment (reference points 2-1). The prolonged drought stress lasting 10 days affected transcriptomes of both genotypes studied more drastically and the number of DEGs was similar in both genotypes (reference point 3-1; respectively, 1,153 Sebastian-specific DEGs and 1,102 mutant-specific DEGs). However, the analysis allowed genotype-specific sets of genes that indicated a possible regulatory role of CBP20 in response to water deficit in barley to be distinguished (Figure 9). In order to obtain the biological relevance of genotype-specific DEGs and to gain insight into processes that are affected by drought stress specifically in *hvcbp20.ab*, the enrichment of the functional annotations (GOs) of barley genes was performed using the PLAZA Monocots 3.0 database. The analysis permitted to identify over-represented Biological Processes (BP; corrected *P* < 0.01), which were up- or down-regulated specifically in either the wild-type “Sebastian” or *hvcbp20.ab* mutant (Supplementary Materials S7, S8).

**Figure 10:**
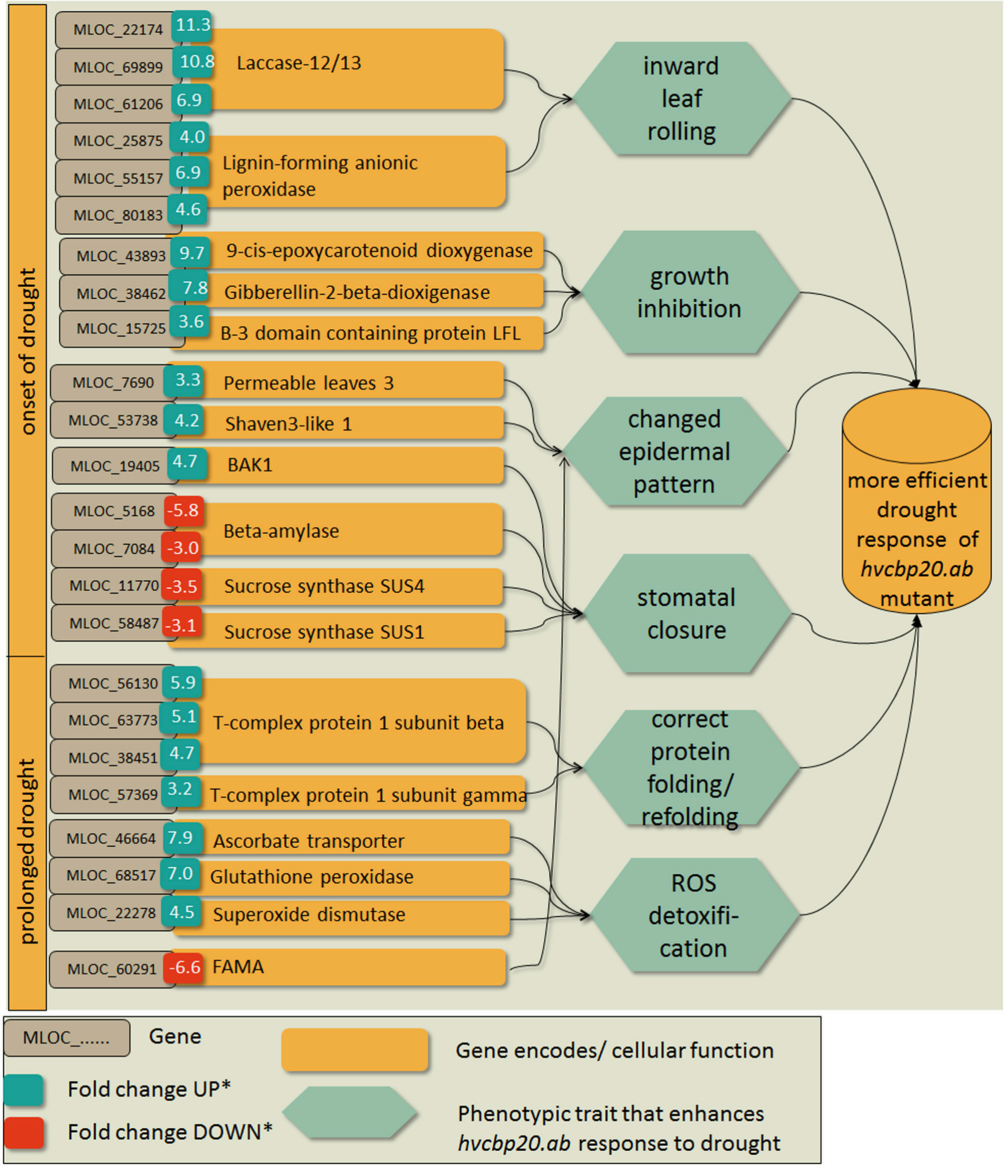
Selected genes that were expressed specifically in the *hvcbp20.ab* mutant at the onset of drought stress and after prolonged stress that might imply the phenotypic traits that enable a better adaptation to stress. Blue boxes indicate upregulation expressed as a fold change of DEG's expression at the onset of drought stress/control and after prolonged drought stress/control in *hvcbp20.ab* mutant.

In response to the decrease in soil moisture at the onset of drought stress, three times more genes were up-regulated in *hvcbp20.ab* than in the WT. The GO enrichment analysis of 429 genes that were specifically up-regulated in the mutant at the onset of drought stress revealed that these genes are involved in four significantly over-represented BPs: the single-organism process, the response to stimulus, the single-organism metabolic process and trichome differentiation (Supplementary Materials S7, S8). Detailed analysis showed that the highly up-regulated genes MLOC_22174, MLOC_69899, MLOC_61206 encoded the enzymes that belong to the plant laccases family and are involved in lignin biosynthesis. Apart from laccases, the up-regulation of genes encoding the lignin-forming anionic peroxidases (MLOC_25875, MLOC_55157, MLOC_80183) was observed in the *hvcbp20* mutant (Figure 10). The analysis of genes up-regulated specifically in mutant at the onset of drought stress revealed also the engagement of phytohormone crosstalk in the *hvcbp20.ab* response to drought stress. At the onset of drought stress, the expression level of the barley gene encoding the 9-cis-epoxycarotenoid dioxygenase (NCED, MLOC_43893) was observed. Interestingly, simultaneously with the up-regulation of the ABA biosynthesis gene, gibberellin 2-beta-dioxygenase (MLOC_38462), which is responsible for the deactivation of bioactive gibberellins, was up-regulated in the *hvcbp20.ab* mutant at the onset of the drought. Moreover, another gene that is involved in the GA/ABA homeostasis encoding the B3 domain-containing protein LFL1 (MLOC_15725; ortholog of Arabidopsis AT3G26790; FUSCA 3) was up-regulated during the phase of a rapid decrease in soil moisture. Also, the level of expression of MLOC_19405 (ortholog to the Arabidopsis gene-encoding BAK1—BRI1-ASSOCIATED RECEPTOR KINASE 1) was increased in the mutant, suggesting the role of brassinosteroids in response of *hvcbp20.ab* to drought stress. Another group of the up-regulated genes at the onset of drought stress that was observed in the *hvcbp20.ab* mutant but not in the WT, was involved in trichome differentiation: MLOC_7690 (PERMEABLE LEAVES3), MLOC_53738 (SHAVEN3-like 1), MLOC_64670 (Protein phosphatase 2A) (Figure 10).

Under the same conditions, the stress response machinery was not activated in the WT to the same extent as in *hvcbp20.ab*. The transcriptome analysis supported our hypothesis that *hvcbp20.ab* sensed the water deficit much earlier and thus was able to adapt to the changed conditions better. At the onset of drought stress, significantly fewer genes were up-regulated in the WT in comparison to the mutant (144 HC genes). GO enrichment analysis revealed the identification of over-represented GO categories including a response to chemical, zinc ion transmembrane transport, de-etiolation, ncRNA processing, a response to metal ion and a response to an inorganic substance (Supplementary Materials S7, S8).

At the onset of drought stress, fewer genes were down-regulated than were up-regulated in *hvcbp20*.ab (504 and 629 DEGs, respectively; Figure 9). Among the 251 DEGs that were specifically down-regulated in the mutant, the most enriched group of genes was annotated as transport and carbohydrate metabolic processes BP categories (Supplementary Material S8). A detailed analysis of the genes classified into these BP categories revealed the barley genes involved in (i) starch metabolism—MLOC_5168 and MLOC_7084 encoding orthologs of Arabidopsis: *BETA-AMYLASE 5* (AT4G15210) and *STARCH-EXCESS 4* (AT3G52180), respectively and (ii) sucrose metabolism (MLOC_11770 and MLOC_58487; Figure 10).

The analysis of the down-regulated genes at the onset of drought stress in the WT revealed the negative influence of a rapid decrease in soil moisture on the “Sebastian” metabolism. The GO enrichment analysis of 196 genes that were specifically down-regulated in the WT allowed the DEGs to be classified into eight BP categories. Among these were photosynthesis, glycolysis and the regulation of developmental growth (Supplementary Materials S7, S8). However, most of down-regulated genes were annotated as being involved in the photosynthesis process. Other highly enriched BPs among the down-regulated transcripts in the WT were the cellular carbohydrate metabolic process and glycolysis, which are related to a reduction in photosynthesis. At the onset of drought stress, another enriched biological process was the regulation of developmental growth. The down-regulation of the genes involved in the growth processes was highly correlated with the observed rapid reduction in the height of the WT plants that were exposed to drought stress (Table 2).

The exposure to prolonged drought stress showed the more complex mechanism of coping with a water deficit in *hvcbp20.ab*, thus stressing the regulatory function of *HvCBP20*. An analysis of the DEGs that were specific to *hvcbp20.ab* after 10 days of severe drought revealed the up-regulation of 591 and the down-regulation of 511 HC genes. Under the same conditions, the majority of DEGs that were specific for the WT were down-regulated (866 genes) whereas only one third of all of the DEGs (287 genes) that were specific for the WT appeared to be up-regulated (Figure 9).

The up-regulated genes specific for the WT were annotated as the small molecule catabolic process, protein import, the cellular amino acid catabolic process, the branched-chain amino acid metabolic process, the leucine metabolic process and mRNA cleavage.

An analysis of the GO annotations of the genes that were significantly up-regulated in *hvcbp20.ab* by severe drought showed their engagement in the following over-represented BPs: protein folding and the cellular response to stress (Supplementary Materials S7, S8). Plants are able to refold the misfolded proteins using chaperonins such as the TCP-1/cpn60 chaperonins (MLOC_56130, MLOC_63773, MLOC_38451, MLOC_57369) that were up-regulated in the *hvcbp20.ab* mutant (Figure 10). Taking into account the metabolic changes in the *hvcbp20.ab* mutant during prolonged drought exposure, the up-regulation of the genes whose products are involved in Reactive Oxygen Species (ROS) detoxification should be stressed. Among the DEGs in this category are MLOC_46664 (ascorbate transporter LPE1), MLOC_68517 (Glutathione peroxidase) and MLOC_22278 (Superoxide dismutase; Figure 10).

A GO annotation analysis of the subset of down-regulated genes in the WT classified them as being involved in cytokinesis, the microtubule cytoskeleton organization, the carbohydrate metabolic process and the lipid metabolic process (Supplementary Materials S7, S8).

The 10-day water deficiency in the *hvcbp20.ab* mutant led to the down-regulation of a set of genes that is involved in several biological processes such as the single-organism cellular process, the phosphate-containing compound metabolic process and the ion transmembrane transport as the GO analysis revealed (Supplementary Material S7). One of the genes that was involved in the single-organism cellular process was MLOC_43989 encoding a putative ortholog of the Arabidopsis SCARECROW-LIKE PROTEIN 27 (SCL27, AT4G00150; Figure 10). Another highly down-regulated gene of this GO category was MLOC_60291 encoding the putative ortholog of Arabidopsis FAMA (AT3G24140; Figure 10).

#### The set of mutant-specific genes that exhibited a similar pattern of expression at the onset and prolonged drought stress

Although, the analysis of mutant-specific DEGs was performed at the onset of drought and after prolonged drought, we addressed a question whether there is set of DEGs that exhibit the same pattern of expression at the beginning and the end of drought treatment. Comparative analysis of the DEGs upregulated at the onset and after prolonged drought stress specifically in mutant revealed the set of 172 genes characterized by the same pattern of expression. Similarly, analysis performed in the case of downregulated genes revealed the set of 87 genes that persisted downregulated during the experiment (Supplementary Material S8). The GO enrichment analysis of genes upregulated in mutant during the whole drought treatment led to the identification of over-represented GO categories such as: root meristem growth, negative regulation of gibberellin biosynthetic process, regulation of auxin polar transport and positive regulation of abscisic acid biosynthetic process. The set of downregulated genes showed enrichment in BP categories related to carbohydrate metabolic process.

**Figure 11:**
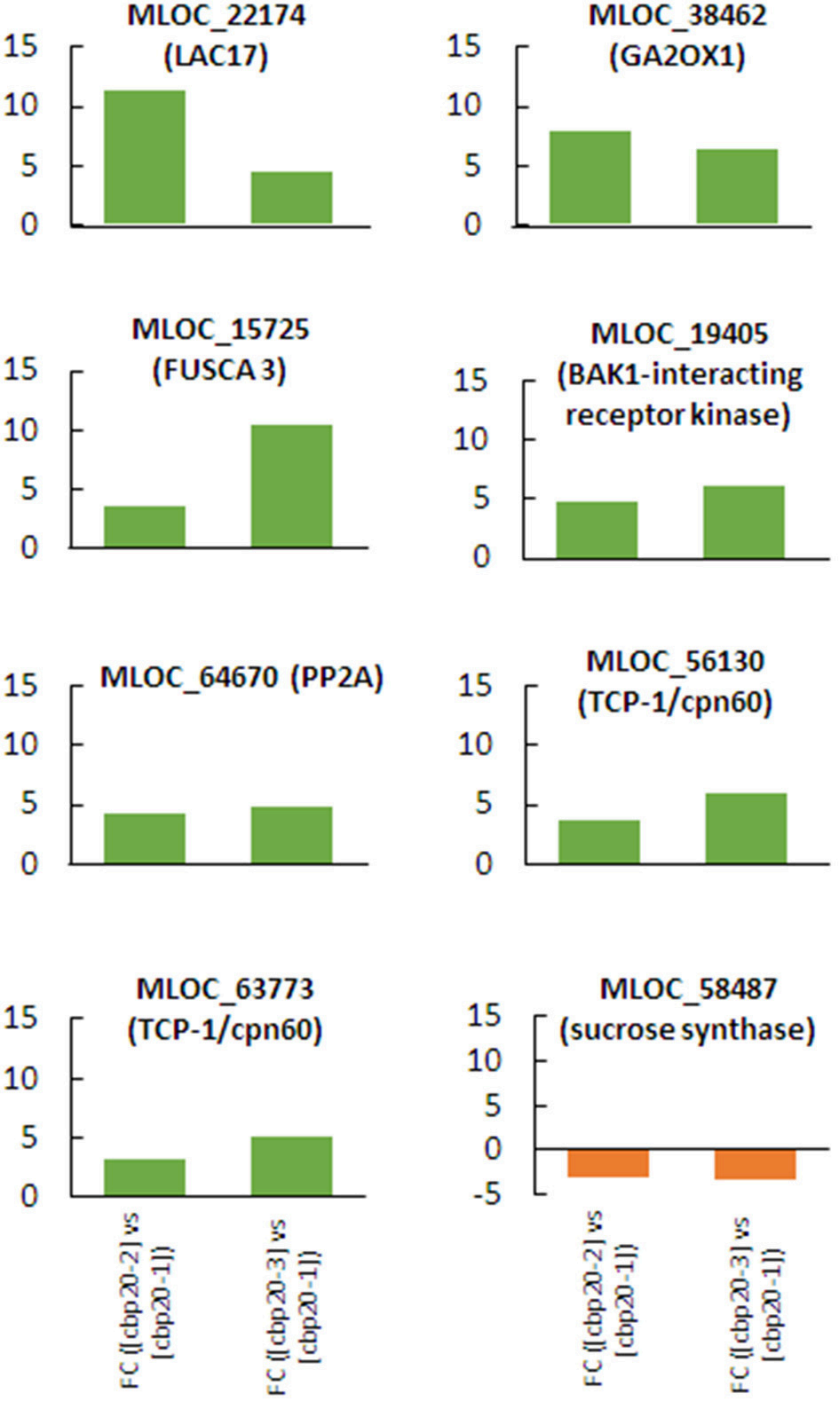
The profiles of expression of selected genes that exhibited the same pattern of expression (up- or down-regulation) during all phases of drought experiment in *hvcbp20.ab*.

Further analysis of DEGs that persist through whole drought treatment with similar pattern of expression specifically in mutant was aimed to the set of genes that might imply the phenotypic traits that enable a better adaptation to stress, based on previously described analyses. It was found that eight from 24 selected genes (Figure 10) had similar pattern of expression during the progression of drought treatment in the mutant (Figure 11).

## Discussion

The huge potential of CBP20 (Cap Binding Protein 20) as a regulatory master that coordinates many processes was pointed since its role in regulation of miRNA biogenesis and the regulation of alternative splicing events has been documented (Kim et al., 2008; Laubinger et al., 2008). Here, we report the comprehensive transcriptome profiling together with phenotyping under drought stress conditions of newly identified barley mutant in *CBP20* (*Cap Binding Protein 20*) gene in order to elucidate its role in barley response to drought.

### Adaptome of *hvcbp20.ab*—the initial response of the *hvcbp20.ab* transcriptome at the onset of drought stress

At the onset of drought stress the up-regulation of genes involved in lignin biosynthesis was observed. Interestingly, the expression of plant laccases, which correlates with the lignification process, is induced in response to biotic and abiotic stresses (Moura et al., 2010; Zhao et al., 2013; Eudes et al., 2014). In tomato and maize roots, the transcription of a laccase gene was induced by salt stress (Wei et al., 2000; Liang et al., 2006). Moreover, the overexpression of a putative laccase gene from rice, *OsChI1*, increased the tolerance of transgenic Arabidopsis to drought and salinity (Cho et al., 2014). Apart from laccases, peroxidases are another class of enzymes involved in the oxidation of lignin precursors (Zhao et al., 2013) that were upregulated specifically in *hvcbp20.ab* mutant. In monocots, highly lignified sclerenchyma cells participate in the process of rolling the leaf inwardly in response to drought stress that enhances the tolerance to water deficit stress (Kadioglu et al., 2012). The up-regulation of lignin-forming enzymes can partially explain the leaf rolling phenotype that was exhibited by *hvcbp20.ab* at the onset of the drought treatment. Leaf rolling in response to a water deficit protects plants from the hazardous effects of stress by decreasing water loss (Kadioglu et al., 2012; De Souza et al., 2013; Terzi et al., 2013). It can be assumed that the ability of *hvcbc20.ab* to store a higher content of water within the leaf after drought stress can also be an effect of leaf rolling that occurred in *hvcbp20.ab* much faster in response to a water deficit than in the WT. The first visible signs of leaf rolling were documented on 18 DAS (Supplementary Material S2A). Therefore, at the early stage of drought treatment, the mutant had already the reduced evaporation area, whereas in the WT the leaf rolling occurred after a couple of days of severe water deficit. It was reported that various tolerance mechanisms such as the antioxidant system and osmolyte accumulation were induced in the rolled leaves of various plant species under stress conditions (Kadioglu and Terzi, 2007).

Considering the involvement of *HvCBP20* in abscisic acid signaling, it was intriguing to check whether changes in the transcriptome revealed a differential regulation of ABA synthesis. Accordingly, the up-regulation of the gene-encoding enzyme that is engaged in ABA synthesis, 9-cis-epoxycarotenoid dioxygenase (NCED, MLOC_43893), was observed. NCED performs reaction that is considered to be rate limiting in ABA biosynthesis (Tan et al., 2003). Arabidopsis and rice NCED3 expression is highly regulated by abiotic stresses, especially a water deficit (Qin and Zeevaart, 1999; Ye et al., 2011). Simultaneously, the upregulation of genes involved in GA/ABA homeostasis was observed: gibberellin 2-beta-dioxygenase (MLOC_38462) encoding the enzyme responsible for gibberellins deactivation (Richards et al., 2001) and B3 domain-containing protein LFL1 (MLOC_15725; ortholog of Arabidopsis AT3G26790; FUSCA 3). It has already been shown that the genes encoding the dioxygenases are the main sites of the regulation of the GA biosynthetic pathway by environmental signals such as drought stress (Thomas et al., 1999; Magome et al., 2008; O'Neill et al., 2010). FUS3 represses GA biosynthesis through its direct interaction with the promoter of the GA biosynthetic genes, whereas its transient activation during vegetative development increases the ABA levels Nambara et al., 2000; Curaba et al., 2004; Gazzarrini et al., 2004; Lu et al., 2010; Zawaski and Busov, 2014). Another gene involved in phytohormone pathway that was up-regulated specifically in *hvcbp20.ab* mutant was BAK1 encoding the leucine-rich protein kinase interacting with the brassinosteroid receptor (BRI1; Li et al., 2002; Nam and Li, 2002). Recently, it was shown that BAK1 is a positive regulator of stomatal closure under drought conditions through the formation of a complex with OST1 (Open Stomata 1). OST1 displays a dominant kinase activity during the response to drought stress when the ABA signal is relayed to the guard cells. Moreover, the complex formation is enhanced by the increased ABA level (Shang et al., 2015).

The up-regulation of genes involved in trichome differentiation at the onset of drought stress in the *hvcbp20.ab* mutant suggested a potential role of trichomes in the mutant adaptation to dehydration conditions. Interestingly, a significantly higher number of trichomes on both sides of the leaf surface was observed in the mutant compared to the WT in response to drought stress. It should be stressed that the increased number of trichomes was reported to enhance the drought tolerance (Huttunen et al., 2010; Hauser, 2014).

Down-regulated genes specific for *hvcbp20.ab* mutant at the onset of drought stress were categorized as being involved in carbohydrate metabolic process. The stomatal closure in response to water stress is maintained by ion transport but recent evidence also suggests the potential role of sugar metabolism in the regulation of guard cells (Lawson et al., 2008). One of the first steps during guard cell opening is the conversion of starch into disaccharide maltose (reviewed in Daszkowska-Golec and Szarejko, 2013). This reaction is catalyzed by β-amylases (BAMs; Weise et al., 2005). It is worth noting that down-regulation of genes encoding β-amylases was observed in *hvcbp20.ab* mutant. Moreover, the down-regulation of β-amylases transcripts was proved to enhanced drought tolerance through stomatal closure (Prasch et al., 2015). The genes encoding sucrose synthases (MLOC_11770 and MLOC_58487; Figure 10) downregulated in *hvcbp20.ab* mutant were also classified into GO category: carbohydrate metabolic process. The link between guard cell-specific sucrose hydrolysis and stomatal conductance was shown using transgenic potato plants with a silenced *SUCROSE SYNTHASE 3* gene (Antunes et al., 2012). The potato *sus3* mutants exhibited a reduced stomatal conductance and an increased water use efficiency that was related to a decrease in sucrolytic activity. Therefore, the down-regulation of above-mentioned genes can be linked to reduced stomatal conductance observed in *hvcbp20.ab* mutant.

### The response of *hvcbp20.ab* transcriptome to prolonged drought stress

Among the over-represented GO categories when up-regulated genes in mutant were considered, the most attention was attracted by the BP—protein folding since its *p*-value was the highest (*P* = 0.000231). Protein folding plays a pivotal role in the regulation of metabolic processes and stress responses. In plants, the key rate-limiting enzymes and misfolded/damaged proteins are regulated by different strategies and are strongly dependent on environmental cues. The correct folding and subsequent protein assembly is required to ensure the proper functionality of a protein. It was reported that the level of TCP-1/cpn60 chaperonin proteins, which are essential for the correct folding and assembly of polypeptides, increased in response to oxygen radicals (Gatenby, 1992). TCP-1/cpn60 chaperonins (MLOC_56130, MLOC_63773, MLOC_38451, MLOC_57369) were up-regulated in the *hvcbp20.ab* mutant. The correct folding and proper assembly of the TCP-1/cpn60 complex is related to the action of the HSP60 chaperone family (Prasad and Stewart, 1992). Interestingly, two barley genes MLOC_66031 and MLOC_18416 encoding the HSP60 proteins were up-regulated in *hvcbp20.ab* in response to prolonged drought stress. The up-regulation of chaperonin complexes specifically in *hvcbp20.ab* suggests the potential role of *CBP20* as a molecular negative regulator of chaperones during drought stress, but this requires further studies.

Oxygen free radicals are responsible for most of the oxidative damage in biological systems and their deleterious effects on biological structures were described in detail (Asada, 1999; Johnson et al., 2003). Although plants evolved the machinery to cope with oxidation stress, under intense stressful conditions, a cascade of molecular events often results in cell death due to severely damaged target molecules. It should be noted that turning on the genes encoding the enzymes responsible for ROS detoxification at a high level in the *hvcbp20.ab* mutant [MLOC_46664 (ascorbate transporter LPE1), MLOC_68517 (Glutathione peroxidase) and MLOC_22278 (Superoxide dismutase)] but not in its WT supports our hypothesis on the better adaptation of *hvcbp20.ab* to drought conditions. These results clearly showed that the mutant developed the ability to protect itself from the deleterious effects of the decreased photosynthesis efficiency under drought. Interestingly, one of the mentioned genes, MLOC_22278, encodes the barley ortholog gene of Arabidopsis *CSD1* (copper/zinc superoxide dismutase 1) that is under miR398 regulation (Sunkar et al., 2006). It was shown that miR398 under drought stress was down-regulated in Arabidopsis and thus *CSD1* was up-regulated in order to perform its protective function (Sunkar et al., 2006). miR398 and its target sites on *CSD1* and *CSD2* mRNA are conserved in dicotyledonous and monocotyledonous plants (Bonnet et al., 2004; Jones-Rhoades and Bartel, 2004; Sunkar et al., 2005). The most striking observation was the fact that miR398 is one of miRNAs that is related to CBP20/CBP80 (CBC) action during miRNA biogenesis (Laubinger et al., 2008). It can be hypothesized that this process is conserved across species as our observations have indicated the indirect involvement of CBP20 in the mechanisms that are connected with ROS detoxification under drought stress.

Among genes that were down-regulated specifically in *hvcbp20.ab* after prolonged drought treatment was MLOC_43989 encoding SCARECROW-LIKE PROTEIN 27 (SCL27, AT4G00150). Recently, the link between SCL27, miR171 and chlorophyll *a* biosynthesis was reported in Arabidopsis (Ma et al., 2014). It was shown that SCLs are down-regulated by miR171 and that it is also able to repress the expression of the gene encoding protochlorophyllide oxidoreductase (POR; Ma et al., 2014). Interestingly, Laubinger et al. (2008) determined that the biogenesis of miR171 was under the control of CBC.

Another highly down-regulated gene in *hvcbp20.ab* mutant after 10 days of drought stress was the putative ortholog of Arabidopsis FAMA (AT3G24140), whose activity is required to promote the differentiation of the stomatal guard cells and to halt the proliferative divisions in their immediate precursors (Ohashi-Ito and Bergmann, 2006). It is worth noting that the function of FAMA is conserved across species including both di- and monocotyledons plants (Liu et al., 2009). Interestingly, a detailed analysis of the changes in MLOC_60291 expression revealed its higher level in control conditions compared to the WT (Supplementary Materials S7, S8) and at the onset of drought in *hvcbp20.ab* (Figure 10). Its expression decreased in response to prolonged drought stress in the mutant. These data provide a partial explanation of the epidermal pattern that was observed in *hvcbp20.ab*. The higher number of guard cells in the mutant compared to WT may be associated with the higher level of FAMA expression during development under an optimal water supply. On the other hand, after drought treatment, clusters of guard cells that were not spaced properly were detected and this could be interpreted as the effect of a strong inhibition of FAMA expression during a prolonged drought. Both types of regulation seemed to help *hvcbp20.ab* in its more efficient adaptation to the changed water status and enabled its proper response to stress.

There was also a group of DEGs that showed the same pattern of expression at the beginning and the end of drought treatment within the set of genes that might imply the phenotypic traits of *hvcbp20.ab* that enable a better adaptation to stress. It strongly support our hypothesis about the involvement of CBP20 in regulation of processes related to (i) lignification (MLOC_22174) and thus possibility of inward leaf rolling; (ii) growth inhibition via hormonal crosstalk (MLOC_38462, MLOC_15725); (iii) stomatal closure (MLOC_19405; MLOC_58487); (iv) changed epidermal pattern (MLOC_64670); (v) correct protein folding (MLOC_56130, MLOC_63773; Figures 10, 11).

### Conclusions and the anticipated mechanism of *hvcbp20.ab* regulation in response to a water deficiency

The response to drought stress at the onset of the water deficit was much faster in the *hvcbp20.ab* mutant than in the WT. The mutant exhibited a better fitness to stress conditions by its much more efficient activation of stress-preventing mechanisms that further enabled its better performance under drought stress.

In summary, compared to the WT, the *hvcbp20.ab* displayed:

1. a reduced growth rate in optimal water conditions and a less rapid inhibition of the growth rate under stress, probably as a result of the interaction between ABA and GA, which was revealed by transcriptome analysis;
2. rapid stomatal closure at the onset of drought stress, which was revealed by both through the assessment of stomatal conductance and transcriptomic analysis;
3. the earlier inward leaf rolling that together with the changed epidermal pattern enabled the formation of a humid environment inside the rolled leaf. This permitted minimal transpiration even under drought stress conditions without a drastic water loss. These phenotypic traits were associated with changes in the expression of the genes encoding the laccases and peroxidases that are engaged in the lignification process, together with the genes that are responsible for guard cell and trichome morphogenesis;
4. an earlier shift of basic metabolism, which was manifested by the better adaptation of the photosynthetic apparatus;
5. the more efficient activation of the protective processes such as protein folding and ROS-scavenging under water deficit conditions, which was revealed by transcriptomic analysis.

All of the above-mentioned traits allowed *hvcbp20.ab* to optimize growth in response to a water shortage and further to withstand a prolonged drought. At present it is not possible to point the direct interactors of CBP20 based on our study, and to define the exact role of cap binding proteins in drought stress response. Instead, we can rather propose the network hubs involved in the adjustment to the drought conditions of *hvcbp20.ab* mutant based on the integration of transcriptomic and physiological data (Figure 10).

It is worth noting that transcriptomic data from Arabidopsis *cbp80* mutant where the significant changes in expression was observed only after ABA treatment (Kuhn et al., 2008) clearly support our results and raise questions about the exact role of CBP20/80 in ABA-related drought response. The most convincing mechanism of action is that expression of genes involved in ABA signaling and ABA-related drought response is regulated by the action of CBP20/80 only in the presence of stress. It is also becoming clear that the mechanism of CBP20 action is selective and does not result in severe phenotype change. Simultaneously, it should be noted that the observed pleiotropy in phenotype is probably due to the regulation of RNA-metabolism.

We believe that results presented here are a key in progress of understanding the involvement of CBP20 in drought stress in barley, we underline that there is still an open question about the exact way of CBP20 action. Further studies will allow to point the genes, that are under direct control of CBP20 regulation.

## Author contributions

ADG designed the experiments, carried the drought stress experiment, generated the physiological data, analyzed and interpreted the microarray data, interpreted the morphological, physiological and molecular results, conducted the *in silico* analysis of the genomic and protein sequence of HvCBP20, performed the 3D modeling of the HvCBP20 protein and wrote the manuscript; AS and MK performed the drought experiment and prepared the material for the microarrays; MS performed the image analyses; MM performed the SEM analyses; MG and PG performed the TILLING screen; TP performed the analysis of the ABA level; KS participated in the photosynthesis analysis; AP and ZSK identified the *HvCBP20* genomic sequence; IS designed the study, contributed to the development of the TILLING mutants and participated in writing the manuscript.

### Conflict of interest statement

The authors declare that the research was conducted in the absence of any commercial or financial relationships that could be construed as a potential conflict of interest.

## Supplementary material

Supplementary Material S1 The analysis of HvCBP20 protein structure. **(A)** The alignment of HvCBP20 protein with its orthologs. Homo, *Homo sapiens*; Macaca, *Macaca fascicularis*; Pteropus -*Pteropus vampyrus*; Elephantulus-*Elephantulus edwardii*; Pan, *Pan troglotydes*; Drosophila, *Drosophila melanogaster;* Hordeum, *Hordeum vulgare*; Oryza, *Oryza sativa*; Brachypodium, *Brachypodium dystachyon*; Sorghum, *Sorghum bicolor*; Zea, *Zea mays*; Arabidopsis –*Arabidopsis thaliana*; Lotus, *Lotus japonicas*; Solanum, *Solanum tuberosum*; Vitis, *Vitis vinifera*. RNP2 and RNP1 (RNA binding domain) depicted as blue boxes; NLS (Nuclear Localization Signal) depicted as red boxes; highly conserved plant-specific domains with unknown function depicted as green boxes. **(B)** Computationally predicted additional H-bond in protein of *hvcbp20.ab* mutant.

Supplementary Material S2 Image analysis of *hvcbp20.ab* and the WT during the drought treatment. **(A)** The phenotype of leaf rolling under subsequent phases of experiment. **(B)** Shoot area of *hvcbp20.ab* and the WT during the drought treatment. The relative reduction of shoot growth of Sebastian and the *hvcbp20.ab* mutant. Percentage of control growth is shown. The gray box behind the charts indicated the drought stress treatment. Statistical analyses were performed using the *T*-test (*P* < 0.01) to assess the differences between genotypes under drought stress. Statistically significant differences are indicated by different lower case letters. Equivalent means have the same letter. **(C)** The illustration of the outcome of the image segmentation process performed using an ImageJ software for color thresholding (Rasband WS, ImageJ, U. S. National Institutes of Health, Bethesda, Maryland, USA, http://imagej.nih.gov/ij/, 1997-2015. Accessed 16 May 2017).

Supplementary Material S3 The content of endogenous ABA in the WT and *hvcbp20.ab* mutant. **(A)** Endogenous ABA level in Sebastian and the *hvcbp20.ab* mutant (μg/ g F_*w*_). **(B)** Endogenous ABA level in Sebastian and the *hvcbp20.ab* mutant expressed as a % of ABA content under control conditions. Statistical analyses were performed using two-way ANOVA (*P* < 0.05) followed by Tukey's honestly significant difference test (Tukey HSD-test) (*P* < 0.05) in order to assess the differences between different water regimes and between genotypes. Statistically significant differences (*P* < 0.05) are indicated by different lower case letters. Equivalent means have the same letter.

Supplementary Material S4 A model illustrating the morphological traits of an *hvcbp20.ab* leaf that play an important role in adapting to drought stress. **(A)** The model of the leaf and epidermal pattern specific for the WT and **(B)** The model of the leaf and epidermal pattern specific for the *hvcbp20.ab* after 10 days of drought treatment.

Supplementary Material S5 Results of the gene expression analysis that was performed using gene-specific qRT-PCR and genome-wide Agilent Barley Gene Expression Arrays of selected DEGs. **(A)** The relative expression of selected genes and **(B)** the correlation between data obtained from Agilent Barley Gene Expression Arrays and qRT-PCR analyses.

Supplementary Material S6 GO categories (Biological Processes) that were over-represented in the *hvcbp20.ab* mutant-specific subsets of genes: down-regulated and up-regulated in control conditions and lists of genes represented by analyzed Gene Ontologies.

Supplementary Material S7 Gene Ontology categories (Biological Processes) over-represented in “Sebastian”- or *hvcbp20.ab* specific subsets of genes, up- or down-regulated during subsequent stages of the experiment in comparison to the control conditions (corrected *P* < 0.01).

Supplementary Material S8 Gene Ontology categories (Biological Processes) over-represented in “Sebastian”- or *hvcbp20.ab* specific subsets of genes, up- or down-regulated (*FC* ≥ 3; *P* ≤ 0.05; FDR) during subsequent stages of the experiment in comparison to the control conditions (corrected *P* < 0.01) and lists of genes represented by analyzed Gene Ontologies.
