# Supplementary figures and images for: Mutation in HvCBP20 (Cap Binding Protein 20) Adapts Barley to Drought Stress at Phenotypic and Transcriptomic Levels
Source: Front Plant Sci. 2017 Jun 2;8:942. doi: 10.3389/fpls.2017.00942 (PMC5454077; doi:10.3389/fpls.2017.00942)

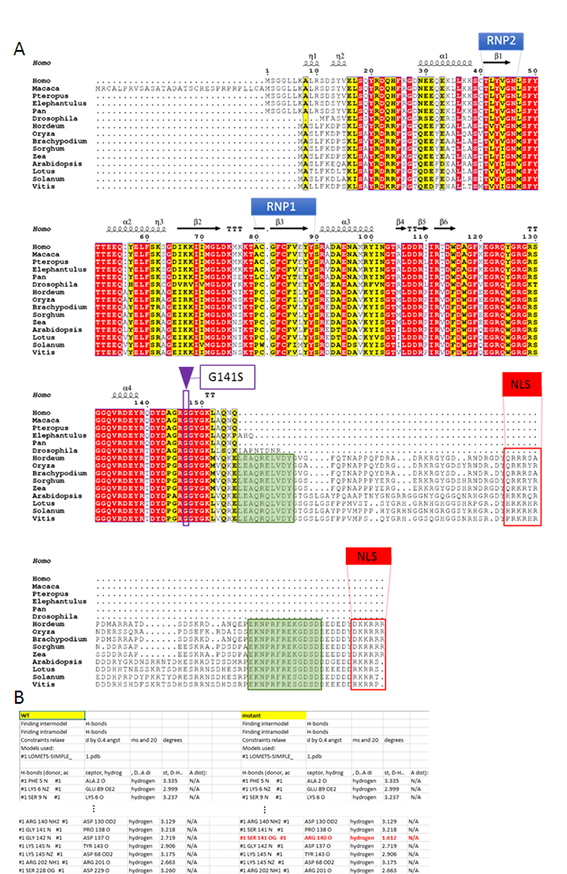

Supplement: Supplementary Material S1 — The analysis of HvCBP20 protein structure. (A) The alignment of HvCBP20 protein with its orthologs. Homo, Homo sapiens; Macaca, Macaca fascicularis; Pteropus -Pteropus vampyrus; Elephantulus-Elephantulus edwardii; Pan, Pan troglotydes; Drosophila, Drosophila melanogaster; Hordeum, Hordeum vulgare; Oryza, Oryza sativa; Brachypodium, Brachypodium dystachyon; Sorghum, Sorghum bicolor; Zea, Zea mays; Arabidopsis –Arabidopsis thaliana; Lotus, Lotus japonicas; Solanum, Solanum tuberosum; Vitis, Vitis vinifera. RNP2 and RNP1 (RNA binding domain) depicted as blue boxes; NLS (Nuclear Localization Signal) depicted as red boxes; highly conserved plant-specific domains with unknown function depicted as green boxes. (B) Computationally predicted additional H-bond in protein of hvcbp20.ab mutant. [file Image1.TIF]

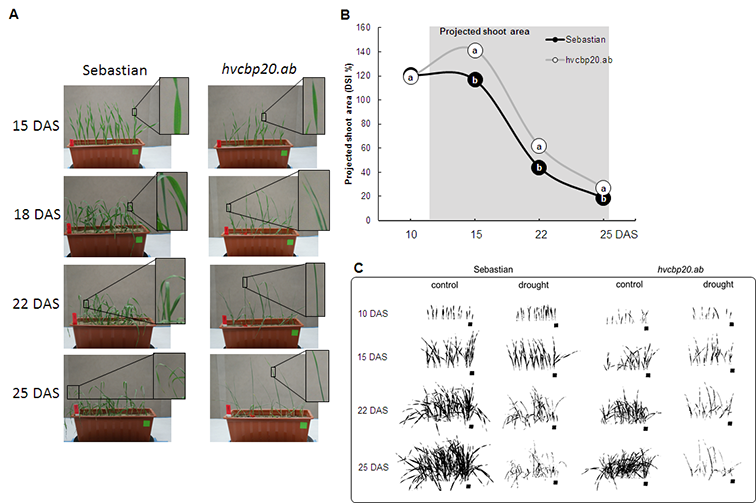

Supplement: Supplementary Material S2 — Image analysis of hvcbp20.ab and the WT during the drought treatment. (A) The phenotype of leaf rolling under subsequent phases of experiment. (B) Shoot area of hvcbp20.ab and the WT during the drought treatment. The relative reduction of shoot growth of Sebastian and the hvcbp20.ab mutant. Percentage of control growth is shown. The gray box behind the charts indicated the drought stress treatment. Statistical analyses were performed using the T-test (P < 0.01) to assess the differences between genotypes under drought stress. Statistically significant differences are indicated by different lower case letters. Equivalent means have the same letter. (C) The illustration of the outcome of the image segmentation process performed using an ImageJ software for color thresholding (Rasband WS, ImageJ, U. S. National Institutes of Health, Bethesda, Maryland, USA, http://imagej.nih.gov/ij/, 1997-2015. Accessed 16 May 2017). [file Image2.TIF]

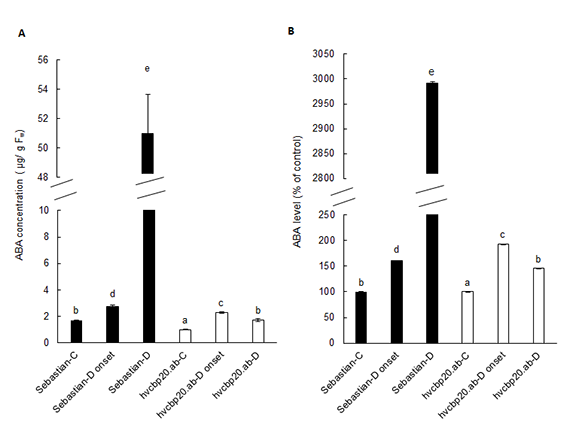

Supplement: Supplementary Material S3 — The content of endogenous ABA in the WT and hvcbp20.ab mutant. (A) Endogenous ABA level in Sebastian and the hvcbp20.ab mutant (μg/ g Fw). (B) Endogenous ABA level in Sebastian and the hvcbp20.ab mutant expressed as a % of ABA content under control conditions. Statistical analyses were performed using two-way ANOVA (P < 0.05) followed by Tukey's honestly significant difference test (Tukey HSD-test) (P < 0.05) in order to assess the differences between different water regimes and between genotypes. Statistically significant differences (P < 0.05) are indicated by different lower case letters. Equivalent means have the same letter. [file Image3.TIF]

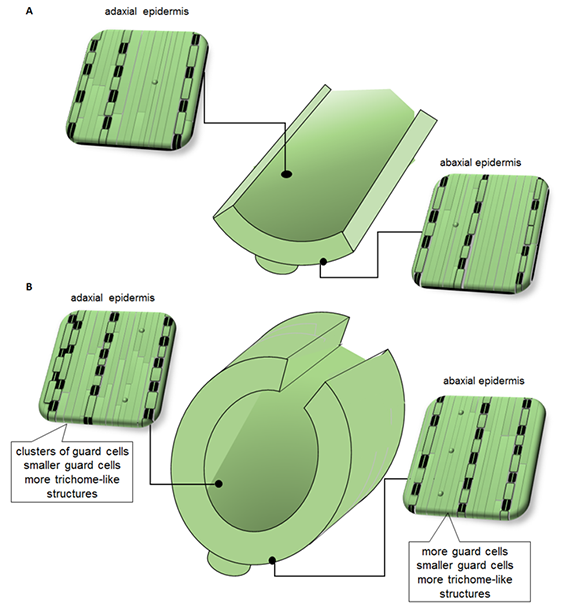

Supplement: Supplementary Material S4 — A model illustrating the morphological traits of an hvcbp20.ab leaf that play an important role in adapting to drought stress. (A) The model of the leaf and epidermal pattern specific for the WT and (B) The model of the leaf and epidermal pattern specific for the hvcbp20.ab after 10 days of drought treatment. [file Image4.TIF]

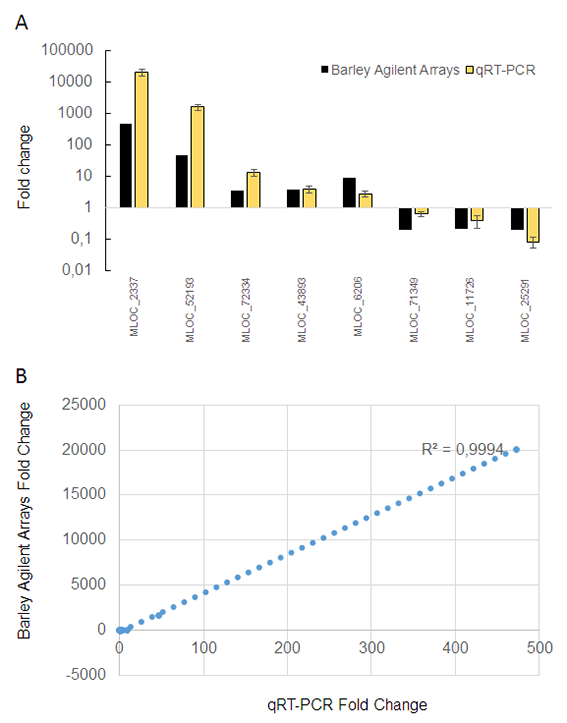

Supplement: Supplementary Material S5 — Results of the gene expression analysis that was performed using gene-specific qRT-PCR and genome-wide Agilent Barley Gene Expression Arrays of selected DEGs. (A) The relative expression of selected genes and (B) the correlation between data obtained from Agilent Barley Gene Expression Arrays and qRT-PCR analyses. [file Image5.TIF]

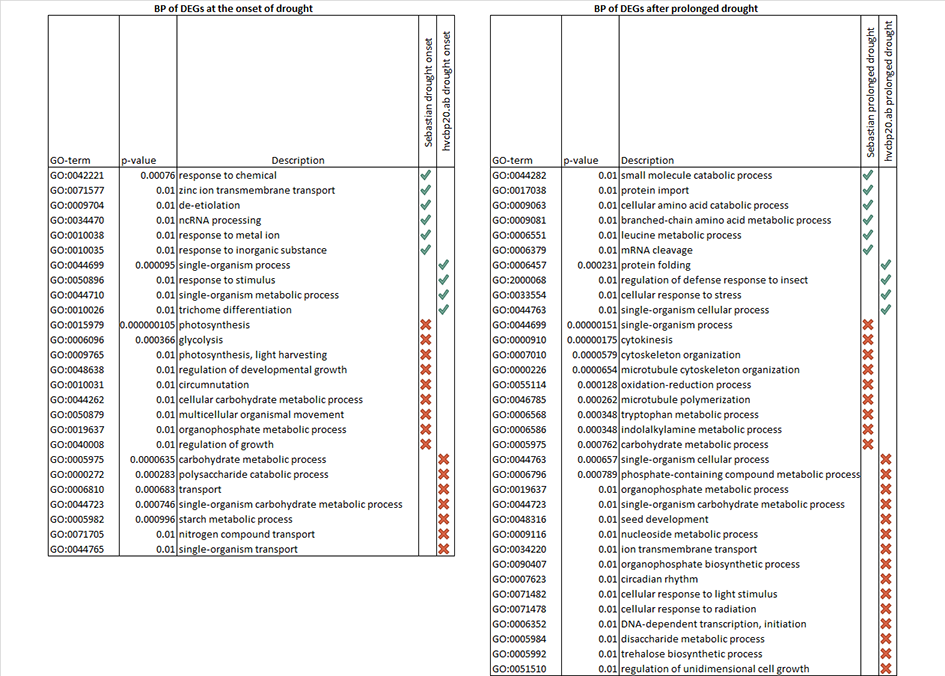

Supplement: Supplementary Material S6 — GO categories (Biological Processes) that were over-represented in the hvcbp20.ab mutant-specific subsets of genes: down-regulated and up-regulated in control conditions and lists of genes represented by analyzed Gene Ontologies. [file Image7.TIF]
